# Responses of Vehicular Occupants During Emergency Braking and Aggressive Lane-Change Maneuvers

**DOI:** 10.3390/s24206727

**Published:** 2024-10-19

**Authors:** Hyeonho Hwang, Taewung Kim

**Affiliations:** Department of Mechanical Design Engineering, Tech University of Korea, Siheung-si 15073, Republic of Korea

**Keywords:** volunteer, evasive maneuvers, midsize male, small female, upright, reclined

## Abstract

To validate active human body models for investigating occupant safety in autonomous cars, it is crucial to comprehend the responses of vehicle occupants during evasive maneuvers. This study sought to quantify the behavior of midsize male and small female passenger seat occupants in both upright and reclined postures during three types of vehicle maneuvers. Volunteer tests were conducted using a minivan, where vehicle kinematics were measured with a DGPS sensor and occupant kinematics were captured with a stereo-vision motion capture system. Seatbelt loads, belt pull-out, and footrest reaction forces were also documented. The interior of the vehicle was 3D-scanned for modeling purposes. Results indicated that seatback angles significantly affected occupant kinematics, with small female volunteers displaying reduced head and torso movements, except during emergency braking with a upright posture seatback. Lane-change maneuvers revealed that maximum lateral head excursions varied depending on the maneuver’s direction. The study concluded that seatback angles were crucial in determining the extent of occupant movement, with notable variations in head and torso excursions observed. The collected data assist in understanding occupant behavior during evasive maneuvers and contribute to the validation of human body models, offering essential insights for enhancing safety systems in autonomous vehicles.

## 1. Introduction

Despite significant advancements in autonomous driving technologies, collisions involving autonomous vehicles (AVs) continue to occur due to factors such as incomplete technological development and mixed traffic conditions involving both AVs and non-autonomous vehicles [1,2]. Numerous studies have examined collisions involving AVs, demonstrating that the risk of collisions persists despite the advancements in autonomous driving technology [3,4,5]. Consequently, continued research into occupant protection in the event of accidents involving AVs remains essential.

Autonomous vehicles (AVs) can perform evasive maneuvers such as emergency braking or lane changes to avoid imminent collisions, but there is still the possibility of collisions occurring [6,7]. In the event of a crash, occupants may face an elevated risk of injury due to being out of the optimal position for which occupant protection systems are designed, resulting from the rapid motion of the vehicle [8]. Therefore, it is necessary to develop occupant protection devices for AVs that consider various occupant positions, not just the proper seating positions optimized for current safety devices.

In fully autonomous vehicles, the role of the driver becomes ambiguous, and the reduction in occupant activity restrictions may lead to substantial changes in the interior and seat configurations [9]. A study exploring preferred seat configurations for autonomous vehicles found that passengers favored seats reclined between 15° and 30° more than the upright configuration for weekend outings or trips [10,11]. When occupants in these reclined seats face evasive maneuvers, their behavior differs from that of passengers in upright seats [12]. Therefore, to ensure the safety of occupants in autonomous vehicles, it is crucial to account for both upright and reclined seating positions.

Anthropomorphic test devices (ATDs), developed to assess the crash safety of traditional vehicles, come in various body types, such as children, small females, midsize male volunteers, and large-sized males [13]. This diversity is essential because interactions with safety devices vary based on occupant anthropometry, resulting in differing levels of protection performance. Initially, crash safety assessments relied solely on 50th percentile male dummies, but it was discovered that smaller females and larger males faced higher risks of injury and death [14]. These variations occur because females are generally smaller and lighter than males, which leads to distinct injury patterns and interactions with restraint systems [15]. Therefore, understanding passenger behavior during evasive maneuvers in autonomous vehicles necessitates studying occupants with various anthropometries.

To evaluate the crash safety of autonomous vehicles, it is necessary to consider various seating configurations, deviations in passenger positions, and diverse body types across multiple crash scenarios. Utilizing numerical techniques is crucial for accommodating a wide range of collision situations, and including the position of displaced passengers at the time of collision is essential for enhancing the accuracy of injury risk predictions. However, current dummies are validated through procedures specialized for specific crash conditions (upright positions in frontal and side collisions) and are predominantly based on cadaver behavior, which has limitations in simulating occupants’ behavior during pre-crash scenarios. Hence, developing an active human body model that can simulate occupant movements during emergency braking or aggressive lane changes is imperative. The development of such an active human body model requires a validation procedure that utilizes test results measuring the behavior of passengers during vehicle evasive maneuvers.

To validate active human body models, studies have been conducted to measure occupant behavior during vehicle evasive maneuvers (Table 1). However, most research has focused on upright positions [16,17,18,19,20] or has not taken into account various occupant sizes [21]. One study measured the behavior of volunteers with body types ranging from the 5th to the 95th percentile under three seat-angle conditions of 23 degrees, 35 degrees, and 47 degrees [12]. However, the study only measured head displacement and lacked data on torso displacement, belt loads, and footrest loads. To the authors’ knowledge, no study has yet considered both midsize males and small females, conducted tests under both emergency braking and lane change conditions in both upright and reclined positions, and measured passenger behavior along with belt load data.

Occupant responses reported in previous studies, such as head excursion, likely varied due to differences in vehicle interior and seat belt geometry, vehicle deceleration pulses, volunteer characteristics, and other factors (Table 1). A couple of studies reported a maximum head displacement of around 210 mm for midsize male subjects using a three-point seatbelt in a 1.1 g braking scenario [17,18]. In contrast, another study found that the average maximum head displacement was 156 mm when the seat was in the middle position and 180 mm when it was moved 75 mm backward [12]. The differences in occupant responses highlight the importance of accurately reconstructing the testing environment, including seat geometry and belt attachment points, which are not publicly available, for validating active human body models against test data. Therefore, documenting occupant responses during evasive maneuvers that consider various types of maneuvers, anthropometries, and seat geometries, along with detailed information about the test vehicle environment, would be beneficial.

The current study aims to examine the behavior of occupants in both upright and reclined postures during crash avoidance maneuvers, including emergency braking and aggressive lane changes. The tests, conducted with small female and midsize male volunteers, were performed in a minivan on a test track. Measurements included vehicle kinematics such as acceleration, linear velocity, and angular velocity, as well as the kinematics of occupants’ heads and torsos, and the interaction forces between the vehicle and occupants. Additionally, the interior of the test vehicle was 3D-scanned to facilitate modeling the test environment, thereby enhancing the usability of the data. This information can improve understanding of occupant behavior during vehicle evasive maneuvers and be used to validate active human body models that simulate passenger behavior in similar scenarios.

## 2. Materials and Methods

### 2.1. Volunteers

The test protocol for this study was determined to be exempt from review by the Ethical Review Board at Tech University of Korea, Siheung-si, South Korea, prior to recruiting volunteers. Following approval, volunteers were recruited through university bulletin boards and social network services. Subsequently, 13 average-build male volunteers with a mean height of 176 cm and weight of 76 kg, and 14 smaller-build female volunteers with a mean height of 155 cm and weight of 51 kg, were enlisted (Table 2). The anthropometric data of the Hybrid III fiftieth percentile adult (H3-AM50) male and fifth percentile adult female (H3-AF5) dummies were also indicated. The target anthropometries of the male and female volunteers were based on those of the 50th percentile adult male and 5th percentile adult female Hybrid III dummies. Initially, the tolerance for the stature and mass were 5 cm and 5 kg, respectively. For female volunteers, it was revised to 8 cm and 8 kg due to difficulty in recruiting the volunteers who met the initial tolerance. Volunteers who satisfied these tolerances were chosen based on a first-come, first-serve basis. Each volunteer was thoroughly briefed on the test procedures and provided consent by signing a form confirming their participation.

### 2.2. Test Vehicle Environment

**Figure 1:**
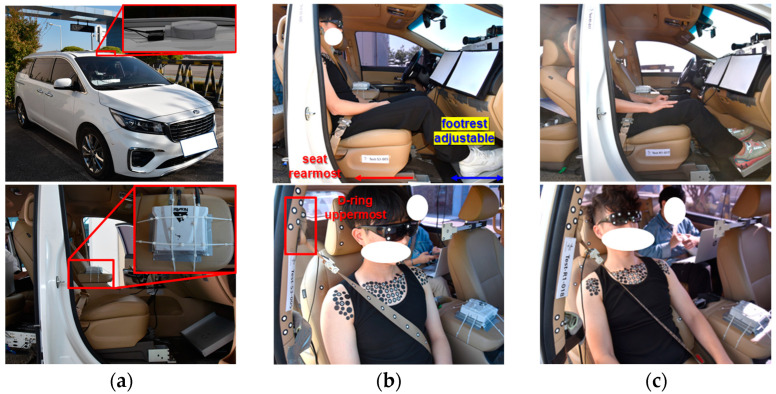
Test vehicle and two seating postures. (**a**) Test vehicle and DGPS; (**b**) upright seatback angle (23 deg); (**c**) reclined seatback angle (43 deg).

A 2019 Kia Carnival minivan served as the test vehicle (Figure 1a). In an autonomous driving setting, the volunteer sat in the front passenger seat, which lacked a steering wheel. The experiments used seatback angles of 23°, known as the upright seatback angle, and 43°, known as the reclined seat angle (Figure 1b,c). These angles were measured using a digital inclinometer at the center of the seatback’s front surface, with manual adjustments in increments of 2–3° to achieve the desired angle. The passenger seat track was adjusted to the rearmost position. Two distinct footrest positions were utilized: one for male volunteers and the other for female volunteers. No fine adjustments were made for individual volunteers. The footrest, sloped at 30 degrees, was selected based on the angle of the passenger-side footrest in the test vehicle. It was constructed from 8 mm-thick aluminum plates, without any paint application. The D-ring of the seatbelt was positioned at the highest setting for all subjects. The lap belt was positioned over the subject’s pelvis and securely fastened. To facilitate modeling the interior of the test vehicle, 3D scans of four conditions—combinations of two seatback angles and two footrest positions—were performed. Scan data, including seat geometry, footrest geometry, belt location, and other relevant details, are provided as Supplemental Materials to this paper.

### 2.3. Vehicle Maneuvers

**Figure 2:**
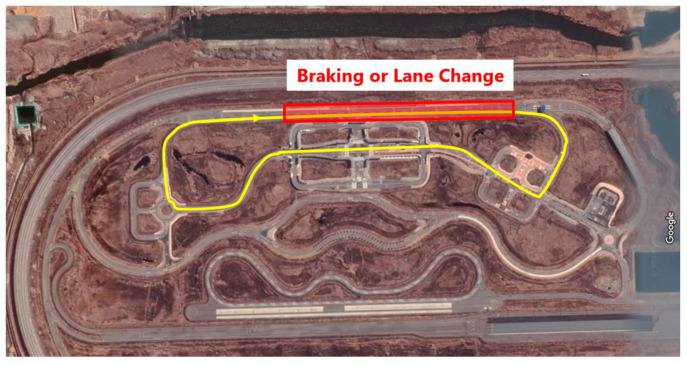
Test vehicle driving path and potential onset area of the evasive maneuvers.

Three types of evasive maneuvers were conducted at the proving ground at the Korea Automobile Test and Research Institute, Hwaseong-si, South Korea (Figure 2). The maneuvers took place on an autonomous vehicle testing track, K-City, offering the volunteer a more realistic experience of road conditions than typical proving grounds. To minimize variations in vehicle kinematics, all tests were driven by a single driver throughout the series.

**Figure 3:**
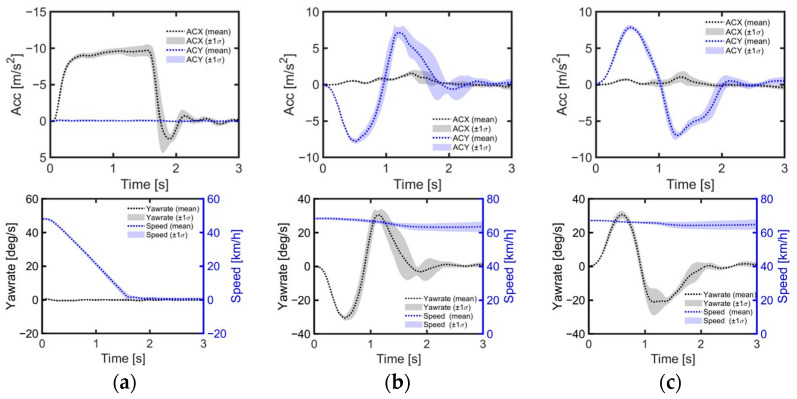
Time histories of vehicle kinematics during evasive maneuvers. (**a**) Emergency braking maneuver; (**b**) left lane-change maneuver; (**c**) right lane-change maneuver.

The emergency braking test involved the vehicle cruising at a steady speed of 50 km/h using cruise control, followed by the test driver abruptly applying maximum braking force, bringing the vehicle to a full stop with a peak deceleration of approximately 0.96 g (Figure 3a). The lane-change tests involved cruising at 70 km/h, followed by a rapid lane change to either side, resulting in a peak lateral acceleration of 0.77 g and a peak yaw rate of approximately 30 deg/s for the initial turn (Figure 3b,c).

### 2.4. Test Protocol

Each volunteer first experienced the braking maneuver with one of two seatback angles, followed by either a left or right lane-change maneuver with one of the two seatback angles; each maneuver was repeated three times (Table 3). For example, a volunteer participated in the braking maneuver with the upright seatback angle, followed by a left lane-change maneuver with the reclined seatback angle. Volunteers were instructed to wear athletic shoes during the test. Volunteers wore the same model of tracksuit pants and sleeveless tops provided by the testing team. This standardized clothing was used to control the coefficient of friction between the seat and the volunteers. The footrest position was adjusted based on the gender of the volunteer. Upon seating, they were asked to sit with their heads in contact with the headrest, which was placed in the lowest position, and to place their hands on their thighs. The test vehicle was driven along the route depicted in Figure 2 one to three times before executing an evasive maneuver for each trial. Until the maneuver, participants were instructed to look forward and relax, either by listening to music through the vehicle’s audio system or engaging in light conversation with the test personnel. No prior instructions were given to the volunteers regarding whether to brace or not during the evasive maneuvers. The evasive maneuver was then unexpectedly performed within the red section, making it challenging for volunteers to predict the onset time of the maneuver in each trial.

### 2.5. Instrumentation

**Figure 4:**
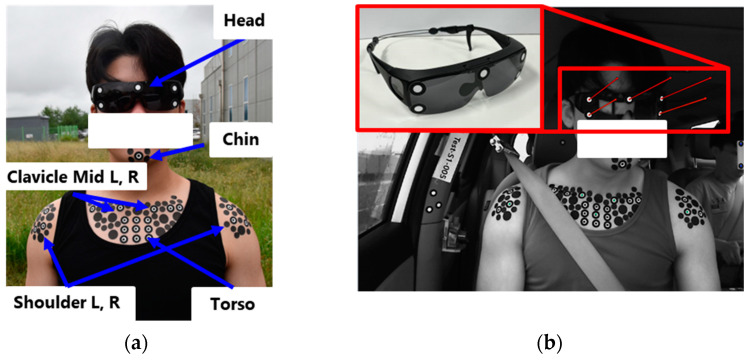
Measurement of occupant excursion. (**a**) markers for stereo-vision motion tracking, (**b**) example of motion tracking results for goggle.

Volunteer kinematics were measured with a stereo-vision motion capture device. Motion tracking markers were attached to the participants’ sunglasses, chin, center of the chest, and shoulder regions (Figure 4a). To minimize relative motion between the sunglasses and participants’ heads, a rubber band was used to secure the sunglasses firmly. These markers were filmed by two high-speed cameras (M-Cam, Nac Image Technology, Tokyo, Japan) mounted on the vehicle’s front dashboard, operating at 100 frames per second. The footage from the cameras was synchronized using a synchronization device (MX-5, Nac Image Technology, Tokyo, Japan). Using the synchronized footage, the displacement and angles of the participants’ heads and torsos were measured with a 3D-marker tracking program (GOM Suite Correlate Pro, ZEISS, Oberkochen, Germany) (Figure 4b). Seat belt load cells (IF966, Humanetics, Farmington Hills, MI, USA) were installed on both the shoulder belt and lap belt before the D-ring and anchor, respectively. The belt load cell had a maximum capacity of 16 kN. To assess its suitability for the main experiment, quasi-static tests were conducted by hanging dead weights of 19, 38, and 55 kg from the end of the instrumented seat belt. The measurement error ranged from 2% to 4%. The belt pull-out amount was measured with a string potentiometer (SP1-25, TE Connectivity, Schaffhausen, Switzerland), positioned parallel to the shoulder belt with its end attached in between the D-ring and belt retractor. To measure the vertical load on the footrest, a load cell (OMEGA85_1900, ATI, Apex, NC, USA) was installed between the footrest and its mounting bracket. The data from these sensors were captured using a DAQ (SCADAS, Siemens, Munich, Germany) at a sampling rate of 3200 Hz. Lastly, the linear acceleration, linear velocity, and angular velocity of the test vehicle were measured using DGPS equipment (PwrPak7-E1, Novatel, Calgary, AB, Canada) mounted atop the central console between the driver and passenger seats (Figure 1a).

### 2.6. Data Processing

#### 2.6.1. Synchronization and Low-Pass Filtering

To synchronize the SCADAS and DGPS devices, satellite times collected from the GPS sensors installed on both systems were utilized. The start point of the evasive maneuver (t = 0) was defined as 0.1 s prior to the instant when the vehicle’s absolute acceleration exceeded 0.1 g in each test. Response corridors were developed using the average and standard deviation values at each timestamp for both volunteers and the vehicle. The test vehicle’s kinematic quantities, such as acceleration, angular velocity, and linear velocity, were filtered using a CFC5 filter [22]. Data from the load cells, including seatbelt and footplate load cells, as well as string potentiometer data, were filtered using a CFC30 filter. The filter classes were lowered from 60 to these values by examining the filtered signals for any unusual high-frequency contents (see Supplementary S1).

#### 2.6.2. Definition of Coordinate Systems

**Figure 5:**
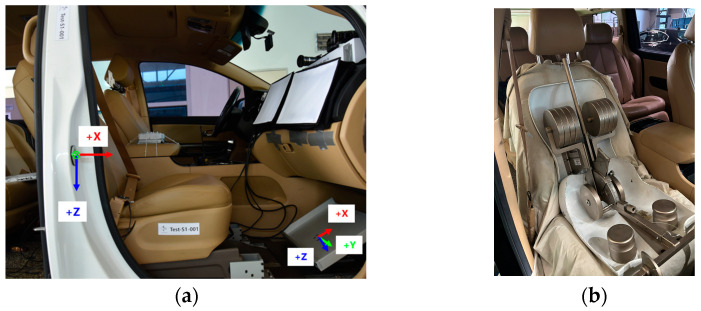
Measurement of occupant excursion. (**a**) Coordinate systems for vehicle and footplate load cell; (**b**) H-point measurement procedure.

The SAE coordinate system designates positive X, Y, and Z directions as forward-moving direction, from left to right seat, and downward direction, respectively. It was adopted to describe the kinematics of both the occupant and vehicle (Figure 5). The origin of the vehicle’s fixed coordinate system was set at the upper bolt of the two bolts on the passenger door striker. The H-point location (X = 101.7 mm, Z = 196.2 mm) for the upright seatback angle was measured to assist in reconstructing the testing environment (Table 3 and Figure 5). The D-ring location relative to the H-point was X = −167.2 mm and Z = −689.3 mm in the vehicle coordinate system (Figure 5). The same D-ring location was used for both male and female volunteers. Note that the H-point measurement device was not available for the reclined seatback condition. The vehicle interior was scanned with a 3D scanner (Leo, Artec 3D, Rue Lou Hemmer, Findel, Luxembourg) featuring motion tracking markers attached to the vehicle interiors. Results from the occupant motion tracking, initially in the coordinate systems of the cameras, were transformed to the vehicle’s fixed coordinate systems using the interior markers.

#### 2.6.3. Body Coordinate Systems

**Figure 6:**
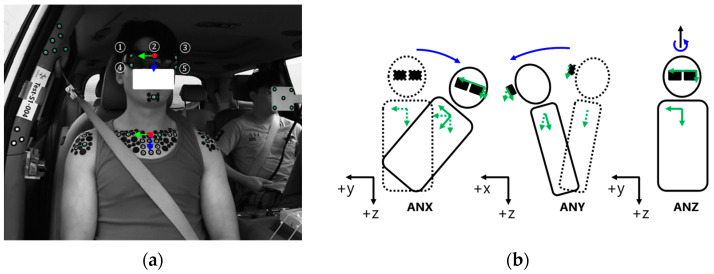
Setting up body coordinate systems. (**a**) head and torso coordinate systems, (**b**) definition of head and torso rotation angles.

To calculate the rotation angles of the volunteers, local coordinate systems were established for the head and torso (Figure 6a). The head’s local coordinate system utilized markers 1, 3, and 4 attached to sunglasses, while the torso’s system was defined using the left and right clavicle markers along with the torso marker. The x-axis was positioned perpendicular to the plane formed by the markers on both the head and torso. The y-axis for the head and torso was established using markers 1 and 3 on the sunglasses and the left and right clavicle markers, respectively. The z-axis was oriented perpendicular to the x- and y-axes. Using these systems, the rotation angles of the head and torso were derived by projecting their axes onto the x-y, y-z, and x-z planes of the vehicle coordinate system (Figure 6b). The angles between the local and global axes were labeled as follows: the angle between the local and global y-axes in the y-z plane was labeled ANX; the angle between the local and global z-axes in the x-z plane was labeled ANY; and the angle between the local and global x-axes in the x-y plane was labeled ANZ.

### 2.7. Sensitivity Analysis

Linear regression analysis was conducted for each maneuver to assess the effects of seatback angles and volunteer stature on the maximum excursions of the head and torso. Up to the first-order interaction term between these variables was considered, along with their main effects. The analysis was performed using Minitab software (version 22.1, State College, PA, USA). To determine which variables to include in the regression equation, the forward option in Minitab was used, with the alpha level for entry set at 0.05. Weight and gender were excluded because these variables were correlated with stature in the current study (Table 2). The peak excursions of the head and torso were extracted from motion tracking data. For the braking maneuver, the maximum forward excursion, considering only the x-component, was analyzed for sensitivity. During the lane-change maneuvers, the lateral excursion, considering only the y-component during the first turn, was analyzed for sensitivity.

## 3. Results

### 3.1. Emergency Braking Maneuver

**Figure 7:**
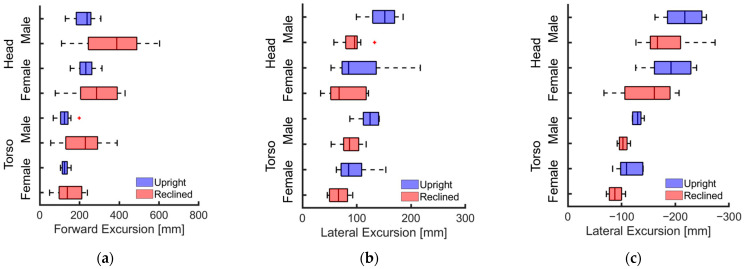
Distribution of head and torso excursions of volunteers during evasive maneuvers. The left and right sides of each box represent the 25th and 75th percentiles, respectively. The vertical line inside the box indicates the median. Whiskers extend from the interquartile range (IQR) to the furthest observation within 1.5 times the IQR. Observations beyond this range are indicated by red cross symbols. (**a**) Braking; (**b**) left lane change; (**c**) right lane change.

**Figure 8:**
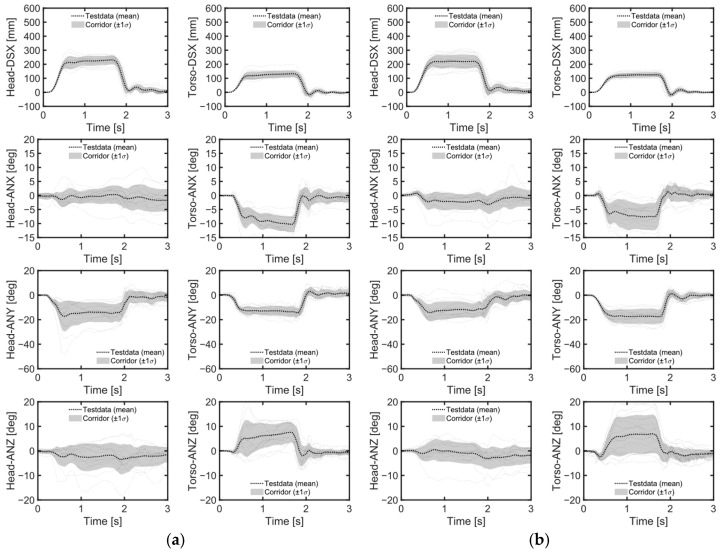
Volunteer response corridor during braking maneuvers. (**a**) Upright male; (**b**) upright female.

**Figure 9:**
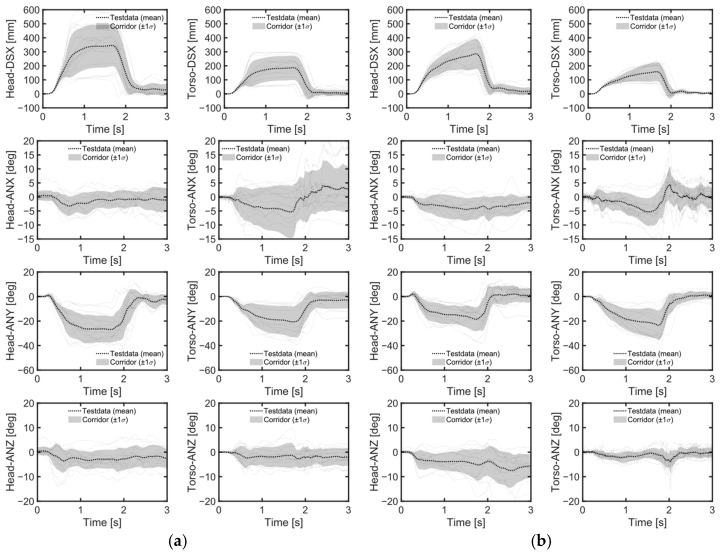
Volunteer response corridor during braking maneuvers. (**a**) Reclined male; (**b**) reclined female.

**Figure 10:**
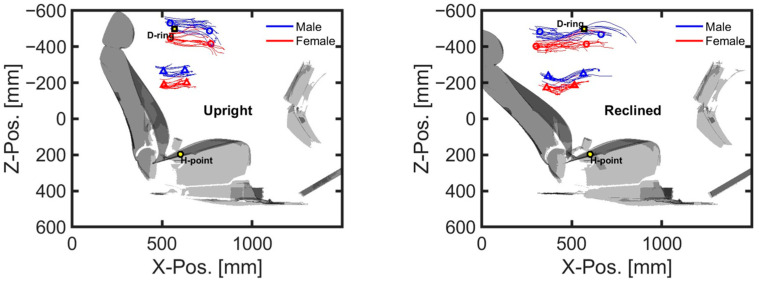
Individual and average head and torso kinematic trajectories during braking maneuvers.

Despite the height differences between male and female volunteers, the mean values and standard deviations of head and chest displacements in the upright position during emergency braking remained similar (Table 4, Figure 7a, Figure 8). However, in the reclined position, the average head displacement for midsize males was 371 mm, which was 89 mm more than that for females (Figure 9). In all instances, the mean values and standard deviations of head and chest displacements were greater in the reclined position compared to the upright position. However, the average furthest forward position of the head occurred in the upright position (Figure 10). Both male and female volunteers showed greater relative head and chest displacements along the x-axis in the reclined position compared to the upright position. The yaw motions of the head and torso differed under upright seatback conditions (Figure 8). The volunteer occupied the first-row passenger seat, causing the shoulder belt to pass over the volunteer’s right shoulder. The torso rotated approximately 9 degrees in the z-direction, advancing the left shoulder forward more due to the shoulder belt’s shape characteristics. In contrast, the head exhibited less rotation in the z-direction than the upper body. In the reclined position, the torso did not rotate along the z-axis as in the upright condition, resulting in minimal differences in z-axis rotation between the upper body and head when reclined (Figure 9).

**Figure 11:**
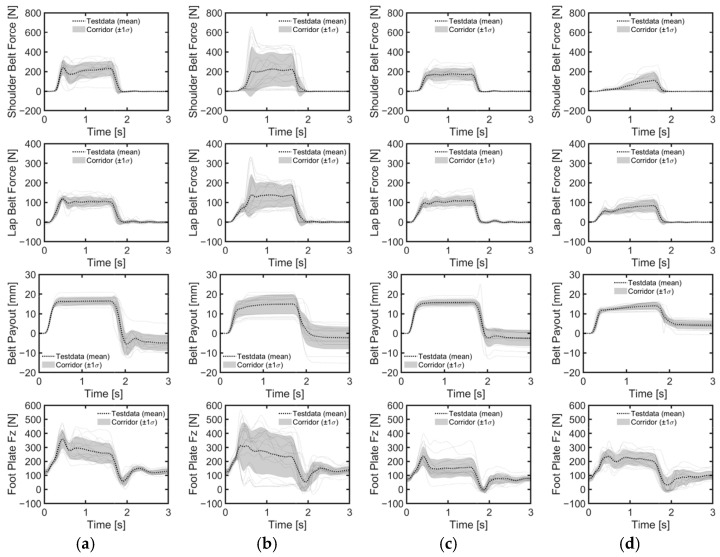
Belt force and foot plate force measured during braking maneuvers. (**a**) Upright male; (**b**) reclined male; (**c**) upright female; (**d**) reclined female.

The reclined position, in comparison to the upright position, resulted in a lower belt load increase rate for both genders (Figure 11). Seatback angle affected belt loads differently between genders. For male volunteers, the standard deviations of both shoulder and lap belt loads increased with seatback angle, while average peak belt loads remained consistent across conditions (Table 4). For female volunteers, the average maximum loads for shoulder and lap belts were reduced in the reclined position compared to the upright position. The load on the footrest remained similar in both positions for all genders, although the standard deviation was higher for male volunteers in the reclined position compared to the upright position. Notably, male volunteers exhibited higher maximum mean loads on the shoulder and lap belts, as well as the footrest, due to their greater weight compared to female volunteers.

### 3.2. Aggressive Lane-Change Maneuvers

During lane changes, the average maximum lateral displacement of both head and chest was 28% greater on average in the upright position compared to the reclined position across all genders (Table 5, Figure 7b,c). Female volunteers, being shorter than male volunteers, experienced an average maximum lateral head excursion that was 14% smaller than that of males across all seatback angles and lane change directions.

**Figure 12:**
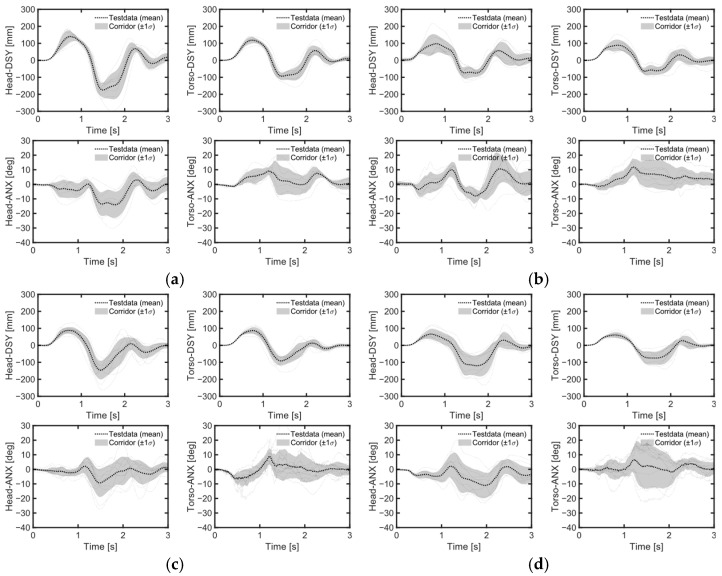
Occupant response during left lane change. (**a**) Upright male; (**b**) upright female; (**c**) reclined male; (**d**) reclined female.

**Figure 13:**
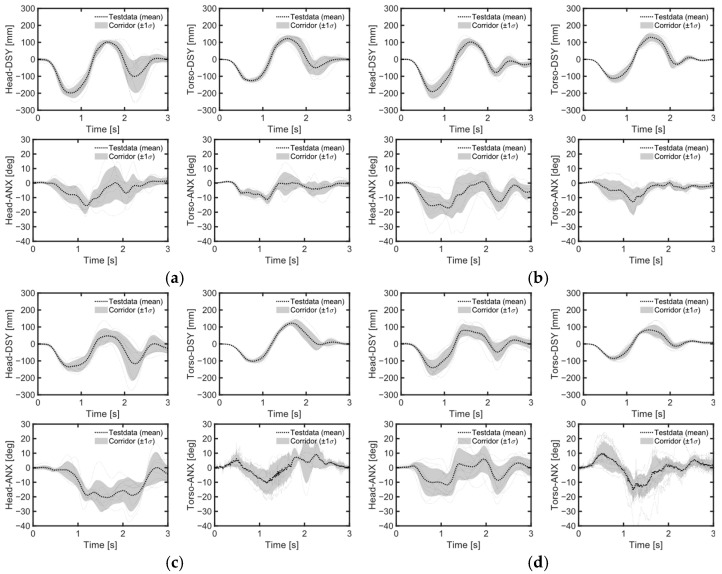
Occupant response during right lane change. (**a**) Upright male; (**b**) upright female; (**c**) reclined male; (**d**) reclined female.

**Figure 14:**
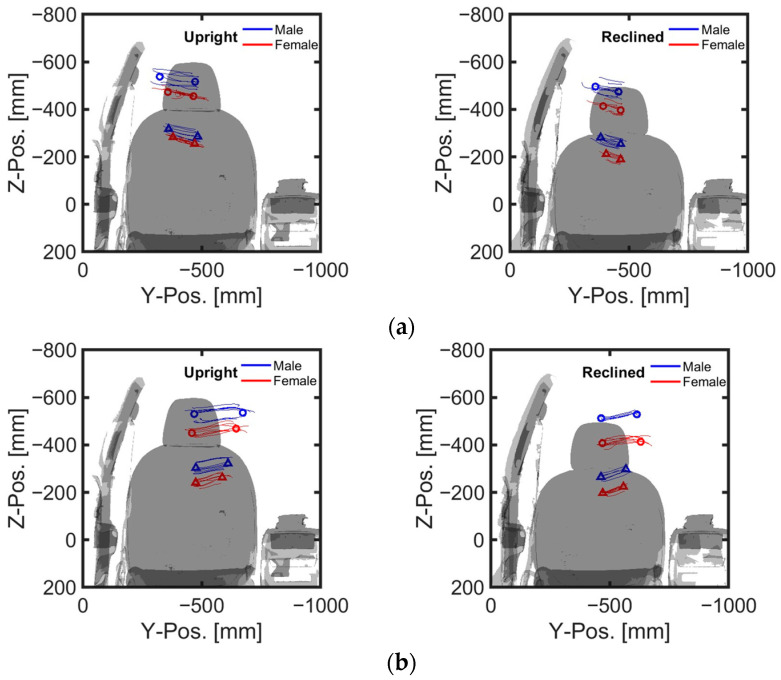
Individual and average head and torso kinematic trajectories in lane-change maneuvers. (**a**) Left lane change; (**b**) right lane change.

**Figure 15:**
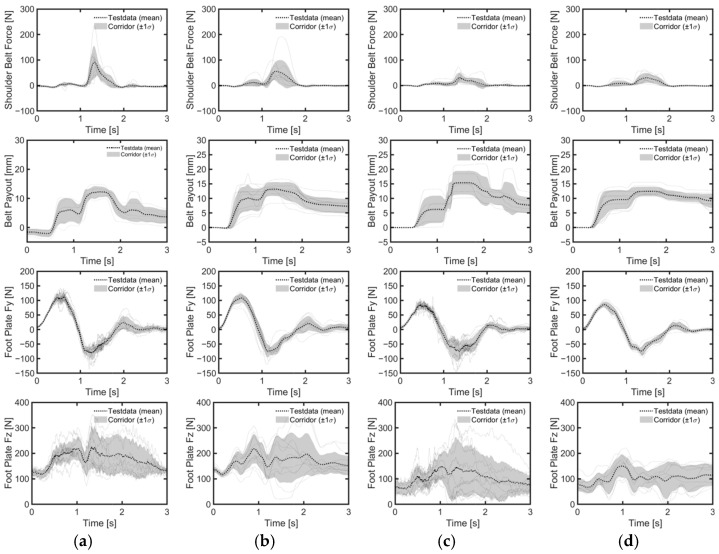
Belt and foot forces during left lane change. (**a**) Upright male; (**b**) reclined male; (**c**) upright female; (**d**) reclined female.

**Figure 16:**
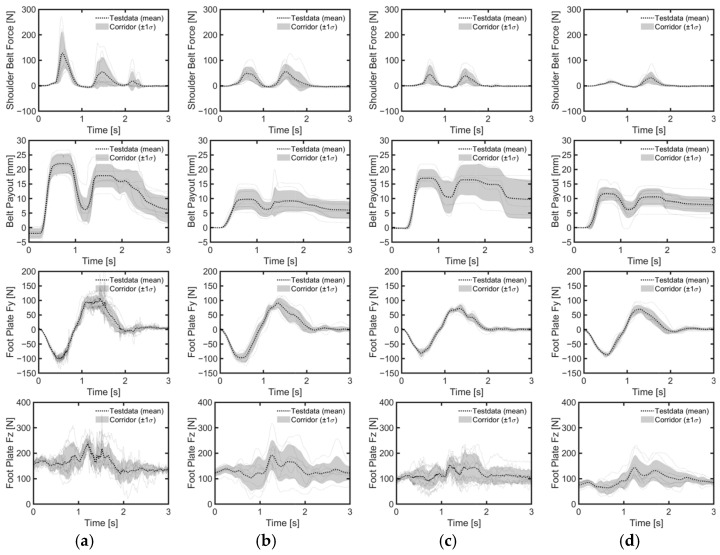
Belt and foot forces during right lane change. (**a**) Upright male; (**b**) reclined male; (**c**) upright female; (**d**) reclined female.

Considering all lane change directions and genders, head displacements were 81% greater during right lane changes compared to left lane changes (Table 5, Figure 7b,c). During right lane changes, subjects moved toward the relatively more spacious center of the vehicle, whereas during left lane changes, they moved toward the vehicle’s door and B-pillar compartment. Another difference observed in the volunteers’ responses between left and right lane changes was the relative displacement of the head and chest. Specifically, during left lane changes, the average peak lateral excursion difference between the head and the torso was 10 mm on average across all genders, while during right lane changes, it was 76 mm on average. Consequently, during right lane changes, the upper body leaned more than it did during left lane changes (Figure 12, Figure 13 and Figure 14). The shoulder belt load was significantly lower during lane-change maneuvers compared to braking maneuvers (Figure 11, Figure 15 and Figure 16). The shoulder belt load remained almost zero until the volunteer reached the maximum excursion point during the left lane change maneuver.

### 3.3. Sensitivity Analysis Results

**Figure 17:**
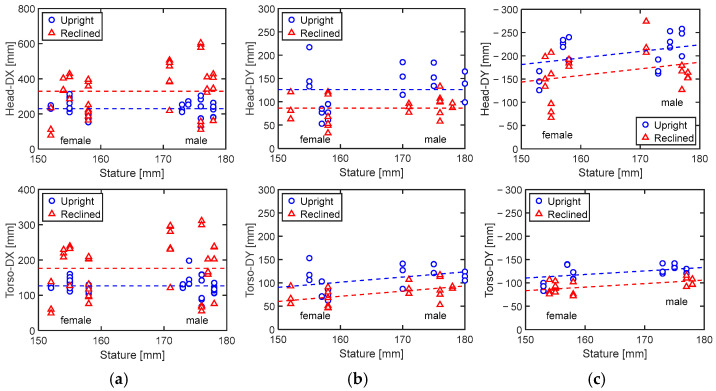
Results of linear regression analysis for maximum excursions. (**a**) Braking; (**b**) left lane change; (**c**) right lane change.

The effect of seatback angle was statistically significant across all three maneuvers (Table 6 and Figure 17). For the lane-change maneuvers, the effect of stature was statistically significant, except for the maximum lateral head excursion during the left lane change. The model fit was better for the lane-change maneuvers compared to the braking maneuver for both head and torso maximum excursions.

## 4. Discussion

### 4.1. Method

This study measured the kinematics of front passenger seat occupants under conditions more reflective of autonomous driving than the driver’s seat, during braking and lane-change maneuvers with both upright and reclined seatback angles. To accommodate varying occupant sizes, midsize adult male and small adult female volunteers were recruited, minimizing the reliance on response scaling techniques. While previous studies have provided valuable insights into occupant responses during evasive maneuvers, many do not include detailed information for reproducing the testing environment, such as interior geometries or belt attachment points [12,16,17,18,19,21]. This is likely because their focus was on exploring effect of parameters such as occupant characteristics, seatback angles, seat tracks, and novel restraint systems on occupant kinematics, rather than primarily on aiding human body model validations. The current study seeks to build on this work by conducting volunteer tests that take into account occupant sizes, seatback angles, and multiple types of maneuvers under a consistent test setup, which was thoroughly documented using 3D scans.

### 4.2. Volunteer Excursions during Emergency Braking

The average maximum excursions of a midsize male seated in an upright position during emergency braking were measured at 227 mm for the head and 126 mm for the torso. This displacement was slightly larger than that observed in previous studies. Specifically, one study reported a maximum displacement of 209 mm for the head and 137 mm for the chest for a similar male subject using a three-point seatbelt in a 1.1 g braking scenario [17]. Similarly, another study observed displacements of 208 mm for the head and 115 mm for the chest under comparable conditions [18]. Moreover, research found that the average maximum head displacement was 156 mm and 180 mm when the seat was in the middle position and when it was moved 75 mm backward, respectively [12]. This indicates that passenger movement increases as the seat is adjusted backward. Additionally, the authors noted that passenger movement during emergency braking was on average 51 mm greater in a minivan compared to a passenger car [12]. These findings suggest that the larger average maximum head and chest displacement observed in this study compared to previous research can likely be attributed to differences in the vehicle’s interior environment. To address this, the location of the H-point and D-ring was measured, and detailed three-dimensional scan data were included as Supplementary Material.

During emergency braking in the upright posture, both male and female volunteers exhibited similar maximum head excursion values (Table 4 and Figure 7a), consistent with previous observations [17]. Notably, the average stature difference between male and female volunteers was 9.4 mm greater in the current study than in the previous study [17], yet both groups demonstrated similar average maximum head excursions. Another also observed that passenger stature did not affect the average maximum head excursion during emergency braking [12]. The volunteers in upright posture reached almost their maximum excursion within approximately 0.5 s of the initiation of the emergency braking (Figure 8). Given the rapid excursions and minimal variation between volunteers, it was hypothesized that the shoulder belt, rather than the volunteers’ muscle control, dictated the maximum excursion. This short duration may have been too brief for volunteers to respond to the sudden braking effectively. Even for the reclined posture, the response corridors were tight, up to 0.5 s, indicating a minor influence of the volunteers’ muscle activity up to that point (Figure 9).

In contrast, for the reclining position, the average maximum head displacement for small female volunteers was 89 mm (24%) less than for midsize male volunteers, while the effect of the stature was not statistically significant (Table 4 and Table 6, Figure 7a). For the volunteer in a reclining position, the displacement gradually increased until between approximately 1 and 1.5 s after braking began and then stabilized (Figure 9). Due to the B-pillar mounted D-ring, the reclined condition resulted in an extended period of occupant excursion. Furthermore, the longer duration of the volunteer’s motion in the reclined condition may be attributed to the center of gravity of the volunteer’s upper body being further back and lower than in the upright position. This could result in a greater counter-moment due to gravity and a smaller moment attempting to rotate the volunteer forward. The less restrictive seatbelt and prolonged event duration led to greater variations among volunteers in the reclined seatback condition than in the upright seatback condition. I was demonstrated that while stature has no statistically significant effect on the forward excursion of the head, the variation in head excursions increases when the seatback is reclined from its upright position [12]. The extended displacement duration in the reclining position highlighted differences in muscle use among volunteers. Therefore, it would be meaningful to validate an active human body model not only for matching the average responses but also for capturing the variations in volunteer responses.

The coefficient of variation for head excursion during braking in the upright posture was around 20% for both genders in the current study (Table 4), whereas the previous studies reported a coefficient of variation of around 30% [12,18]. One possible reason for the smaller coefficient of variation could be the narrower range of stature (± 3 cm) and mass (± 5 kg) among the volunteers in the current study, compared to previous studies with variations of approximately ± 5 cm and ± 10 kg. The more tightly controlled stature and total body mass may have contributed to a more consistent initial occupant posture and belt length, respectively. Additionally, the use of the custom footrest and the instruction given to volunteers to keep their heads in contact with the headrest until the initiation of the maneuver may have contributed to reducing the variation of the initial posture of the volunteer.

### 4.3. Volunteer Excursions during Aggressive Lane Changes

During lane changes, the influence of the seatback angle revealed that the 20 degree reclined conditions from the upright condition resulted in an 11% to 30% smaller average maximum lateral head displacement compared to the upright condition. For all seatback angles and directions, the average maximum lateral head displacement of shorter female volunteers was 7% to 21% less than that of male volunteers (Table 5). It is expected that the smaller displacement in the reclining condition compared to the upright condition is attributed to the passenger’s lower center of gravity when the seat is reclined, leading to less centrifugal force (relative to the vehicle’s coordinate system) for the same lateral acceleration of the vehicle. It was also showed that the increase in the seat reclining angle was associated with the decrease in the lateral head excursion amount in lane-change maneuvers [12]. Moreover, the seats used in the test vehicle were OEM seats that slightly wrap around the sides of the passenger. As the seat reclines further back, the friction between the passenger and the seatback may increase, likely resulting in an enhanced restraint of the passenger’s lateral movement by the seatback. It was also demonstrated that the more the seat wraps around the passenger, the lesser the lateral displacement of the passenger during lane-change maneuvers [18]. Unlike the emergency braking maneuver, there was no clear trend in the variation of the volunteers’ excursions between the upright and reclined seatback conditions during lane-change maneuvers (Table 5).

### 4.4. Occupant Behavior

The difference in rotational behavior between the head and upper body was most pronounced in the upright position. During emergency braking in the upright position, the z-direction rotation of the head (Head-ANZ) was smaller than that of the upper body (Torso-ANZ) for both genders (Figure 7a). In an upright position during emergency braking, the upper body is restrained by the asymmetric shoulder belt, causing the inside (left) shoulder of the passenger-side volunteer to move forward more than the outside shoulder, resulting in positive z-direction rotation of the upper body. Despite the rotation of the upper body, the z-direction rotation of the head remained nearly zero degrees. This likely occurred because the volunteers controlled their posture to maintain a forward gaze during emergency braking.

During the left lane change, the passenger’s head moved outward from the vehicle during the first turn, inward during the second, and finally returned to its original position. In a right lane change, the movement sequence was reversed. Regardless of the sequence, it was observed that volunteers generally tried to tilt their heads inward (towards the center of the vehicle) as they moved outward (or toward the B-pillar) (Figure 12 and Figure 13). Conversely, when their heads moved toward the vehicle’s center, they naturally allowed their heads to tilt in that direction. As a result, for all genders, the average maximum head displacement during a right lane change was 56% to 84% greater in the upright position and 99% to 100% greater in the reclined position compared to a left lane change (Table 5). One study also observed this behavior but did not discuss its underlying causes [19]. One possible explanation for the volunteers’ behavior could be their prior awareness of the distance between their bodies and the vehicle’s interior structure. This directional dependency of head rotation and lateral excursions in the volunteers may indicate that they tried to avoid contact between the vehicle interior and their bodies. By attempting to maintain the initial posture, an active human body model may struggle to capture this type of occupant behavior during emergency maneuvers [23]. Therefore, these results suggests that occupant behavior, including the desire to maintain a forward gaze and avoid contact with the vehicle interior, should be considered when validating active human body model during pre-crash maneuvers.

Lastly, considering the large variations in occupant kinematics and the directional dependency of the volunteers’ behavior during evasive maneuvers, implementing countermeasures to control the occupant’s position within a safe area will be a more efficient approach than developing countermeasures to accommodate various occupant positions.

### 4.5. Limitation

The current study has several limitations worth noting. First, the number of volunteers was small for conducting lane-change maneuvers. Additionally, volunteer recruitment was limited to typical anthropometries commonly used in human body modeling. Conducting further volunteer tests with a more diverse population would be valuable for developing response corridors for a broader range of target populations using functional regression techniques [12,21]. Second, volunteers were exposed to three repeated trials. The repeated test may have influenced the occupant responses. Third, the absence of an anticipated imminent collision may have caused volunteers to brace less than they would in a real-world scenario, potentially increasing the extent of their movements. One study showed that some muscles in the volunteers reached activation levels of up to 30% of their maximum capacity while controlling their postures during the emergency braking maneuver [17]. With a sense of impending danger, volunteers could brace more than they did during the tests. Therefore, it would be valuable to simulate impending danger during pre-crash maneuvers using a driving simulator with a motion platform, virtual reality glasses, or similar technologies [24]. During such experiments, the utilization of cost-effective brain–computer interface (BCI) and eye-tracking systems can provide insight into the volunteers’ reactions to the simulated impending danger [25,26]. Along with electromyography (EMG) sensors, these systems can help us understand the relationship between the perception of impending danger and the occupants’ responses. Fourth, the footrest was used to measure the contact force between the shoe and the footrest surface. The use of the footrest may have affected the kinematics of the volunteers by forcing them to have a certain initial lower extremity posture, which has been shown to have an effect on occupant responses during evasive maneuvers [12]. Fifth, the kinematics of the head and torso were measured using markers placed on the sunglasses worn by the volunteers and directly on their skin. For purposes of model validation, it is necessary to measure the kinematics of the human body model at comparable locations. Lastly, the use of the OEM vehicle complicates the reconstruction of the test environment. To mitigate this issue, a 3D scan of the vehicle interior was included as Supplementary Material. Additionally, the force-deflection characteristics of the seat cushion from the test vehicle are detailed in Supplementary S3.

## 5. Conclusions

The current study analyzed the kinematics of midsize male and small female volunteers seated in the front right seat during evasive maneuvers with typical and reclined seatback angles. The impact of seatback angle and volunteer stature on their kinematics was evaluated.

During the emergency braking maneuvers, both midsize male and small female volunteers with the upright seatback angle exhibited similar maximum forward head excursions. Given the rapid excursions and minimal variation between volunteers, it was hypothesized that the shoulder belt, rather than the volunteers’ muscle control, dictated the maximum excursion. With the reclined seatback angle, the relatively shorter female volunteers exhibited smaller peak excursions than their counterparts. Due to the B-pillar mounted D-ring, the reclined condition resulted in an extended period of occupant excursion. The less restrictive seatbelt and prolonged event duration led to greater variations among volunteers in the reclined seatback condition than in the upright seatback condition. It would be better to validate an active human body model not only by matching the average responses but also by capturing the variations in volunteer responses to investigate potential edge cases.

It seems that the volunteers were not simply trying to maintain their initial postures during evasive maneuvers. Rather, they demonstrated a preference to maintain a forward gaze during emergency braking conditions in an upright posture. In lane-change maneuvers, head excursions and rotations were reduced when volunteers moved toward the door compared to when they moved toward the center of the vehicle, suggesting that volunteers adapt their posture based on the available space during evasive maneuvers. This behavior raises questions about how occupant responses could change if they see actual impending dangers.

The excursion amounts of volunteers in this study differed from those in previous studies, likely due to variations in the test vehicle’s interior environment. This finding underscores the importance of reconstructing vehicle interiors to validate active human models. To facilitate this reconstruction, 3D scan data of the vehicle interior are provided in the Supplementary Material. The results offer crucial measurement data on volunteer behavior during evasive maneuvers in autonomous vehicles, which is essential for developing human models.

Volunteer responses from the current study, along with previous related research, can be used to validate active human body models that simulate occupant responses during pre-crash maneuvers. These models can then be used to develop countermeasures to control the occupant’s position within a safe area during evasive maneuvers. Lastly, the forces measured from the seatbelt and footrest provide insight into the actuation forces required to mitigate occupant excursion during evasive maneuvers.

## Figures and Tables

**Table 1:** Previous studies on volunteer kinematics measurements during evasive maneuvers.

	Maneuver	Volunteer	Seatback Angle	Measurement	Limitation
[16]	Braking	5%tile female, 50%tile female, 50%tlie male	Upright (-)	Head kinematics Torso kinematics	No reclined posture No lane changes
[17]	Braking	50%tile female 50%tile male	22 deg	Head kinematics Torso kinematics Seatbelt load Footplate load Muscle activity	No reclined posture No small female No lane changes
[18]	Braking	50%tile female 50%tile male	14 deg	Head kinematics Torso kinematics Seatbelt load	No reclined posture No small female
	Lane change				
[19]	Lane change	50%tile female 50%tile male	25 deg	Head kinematics Torso kinematics Seatbelt load Footplate load Muscle activity	No reclined posture No small female
[12]	Braking	5%tile~95%tile people	23 deg, 35 deg, 47 deg	Head kinematics	Head kinematics only
	Lane change				
	Turn and brake				
[21]	Braking	50%tile female 50%tile male	24 deg, 48 deg	Head kinematics Torso kinematics Muscle activity	Focused on midsize female No excursion data
	Turn				

**Table 2:** Age, stature, and weight of volunteers and corresponding dummies.

	Female	Male	H3-AM50	H3-AF5
No. of volunteers	14	13	-	-
Age [year]	32 (±10)	27 (±8)	-	-
Stature [cm]	155 (±3)	176 (±3)	175	152
Mass [kg]	51 (±5)	76 (±4)	78	49

**Table 3:** Test matrix for each volunteer.

Maneuver	Seatback Angle [deg]	Awareness of Initiation of Maneuver	Number of Volunteers (Male/Female)	Number of Repetitions
Braking	23	Not informed	12 (6/6)	3
	43	Not informed	13 (7/6)	3
Left lane change	23	Not informed	6 (3/3)	3
	43	Not informed	7 (4/3)	3
Right lane change	23	Not informed	6 (3/3)	3
	43	Not informed	7 (3/4)	3

**Table 4:** Maximum occupant response in braking maneuvers.

Gender	Seatback Angle [deg]	N	Head			Torso			Shoulder Belt Force [N]	Lap Belt Force [N]	Foot Plate Fz [N]
			DSX [mm]	ANY [deg]	ANZ [deg]	DSX [mm]	ANY [deg]	ANZ [deg]			
Male	23	17	227 ± 46	−21 ± 11	−6 ± 5	126 ± 30	−17 ± 3	9 ± 5	282 ± 70	133 ± 18	−372 ± 74
	43	18	371 ± 156	−31 ± 11	−8 ± 4	196 ± 88	−20 ± 12	−5 ± 3	300 ± 222	174 ± 92	−372 ± 121
Female	23	18	233 ± 45	−18 ± 3	−5 ± 4	127 ± 16	−20 ± 6	9 ± 7	206 ± 57	116 ± 28	−251 ± 74
	43	16	282 ± 110	−22 ± 8	−10 ± 7	149 ± 64	−26 ± 11	−6 ± 4	118 ± 84	95 ± 24	−257 ± 52

**Table 5:** Maximum occupant response in lane-change maneuvers.

Maneuver	Gender	Seatback Angle [deg]	N	Head		Torso		Shoulder Belt Force [N]	Foot Plate Fy [N]	Foot Plate Fz [N]
				DSY [mm]	ANX [deg]	DSY [mm]	ANX [deg]			
Left Lane Change	Male	23	9	133 ± 57	6 ± 4	122 ± 18	13 ± 5	106 ± 50	118 ± 11	−249 ± 63
		43	11	93 ± 19	8 ± 4	88 ± 19	12 ± 9	63 ± 48	111 ± 15	−241 ± 75
	Female	23	9	105 ± 52	17 ± 6	94 ± 29	14 ± 6	37 ± 18	87 ± 8	−180 ± 93
		43	9	78 ± 34	8 ± 5	66 ± 18	12 ± 7	35 ± 19	88 ± 9	−160 ± 41
Right Lane Change	Male	23	8	−208 ± 35	−18 ± 4	−131 ± 9	−12 ± 3	136 ± 72	−103 ± 12	−251 ± 35
		43	8	−186 ± 46	−26 ± 7	−105 ± 10	−14 ± 7	86 ± 36	−110 ± 12	−199 ± 27
	Female	23	8	−193 ± 41	−22 ± 9	−115 ± 22	−15 ± 7	54 ± 33	−83 ± 13	−176 ± 46
		43	9	−155 ± 51	−17 ± 7	−88 ± 12	−18 ± 9	35 ± 23	−88 ± 7	−150 ± 47

**Table 6:** Results of linear regression analysis for maximum excursion in mm (only statistically significant variables (*p* < 0.05) were included).

Maneuver	Responses [mm]	Variables			Adjusted R^2^
		Intercept	Seatback [deg]	Stature [cm]	
Braking	Head-DX	115.8	4.96	−	17.30%
	Torso-DX	69.2	2.491	−	14.51%
Left lane change	Head-DY	171.8	−1.990	−	20.85%
	Torso-DY	−40.2	−1.510	1.102	38.91%
Right lane change	Head-DY	−12	1.878	−1.416	21.42%
	Torso-DY	−28.5	1.357	−0.755	58.11%

## Data Availability

Data may be available upon request to interested researchers.

## Supplementary Materials

The following supporting information can be downloaded at: https://www.mdpi.com/article/10.3390/s24206727/s1.
