# Supplementary material for: Responses of Vehicular Occupants During Emergency Braking and Aggressive Lane-Change Maneuvers
Source: Sensors (Basel). 2024 Oct 19;24(20):6727. doi: 10.3390/s24206727 (PMC11511151; doi:10.3390/s24206727)
Supplement: Supplementary file 1 [file sensors-24-06727-s001.zip › sensors-3189024-supplementary.pdf]

1. SUPPLEMENTARY

1.1. SUPPLEMENTARY S1

The effect of the filter class was shown in Figure S1. CFC5 and CFC30 was chosen for DGPS data and SCADAS data, respectively.

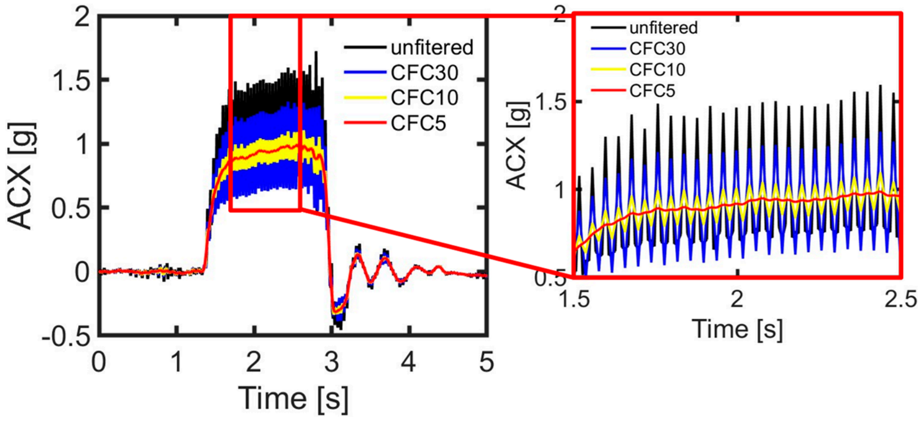

(a) CFC5 (DGPS)

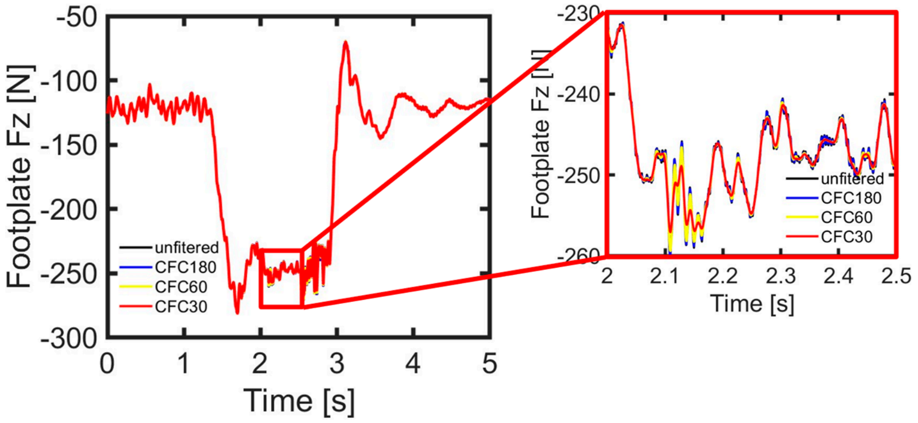

(b) CFC 30 (SCADAS)

Figure S1.1 Effect of the filter class for the raw data

1.2. SUPPLEMENTARY S2

The vehicle and test environment used for measuring the occupant's movement during the autonomous vehicle's avoidance maneuver simulation are presented in the following section. The sensors installed inside the vehicle were placed as shown in Figure S2.1. To provide this internal space information, the test environment for each test condition (Upright Male, Upright Female, Reclined Male, Reclined Female) was scanned using a 3D scanner, and the scanned information is provided as STL files (Figure S2.2, Figure S2.3).

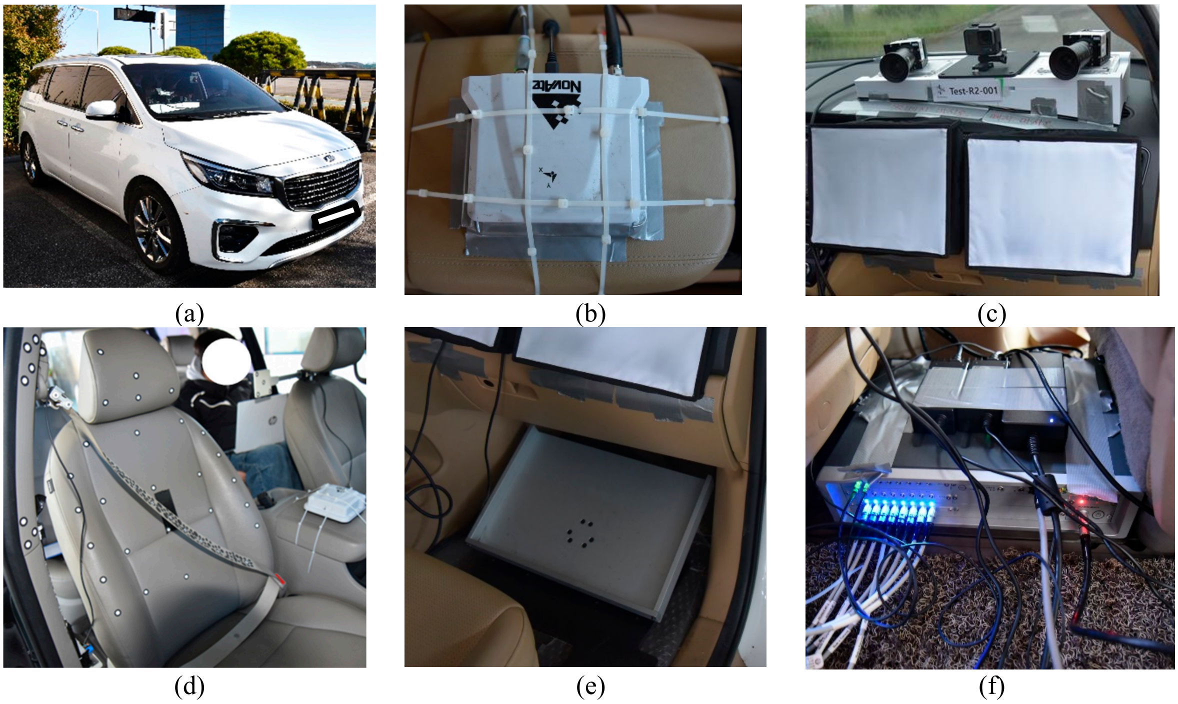

Figure S2.1. Test Vehicle Environment : (a) Test Vehicle, (b) DGPS, (c) Stereo Camera, (d) Belt loadcell and String potentiometer, (e) Foot Plate, (f) SCADAS

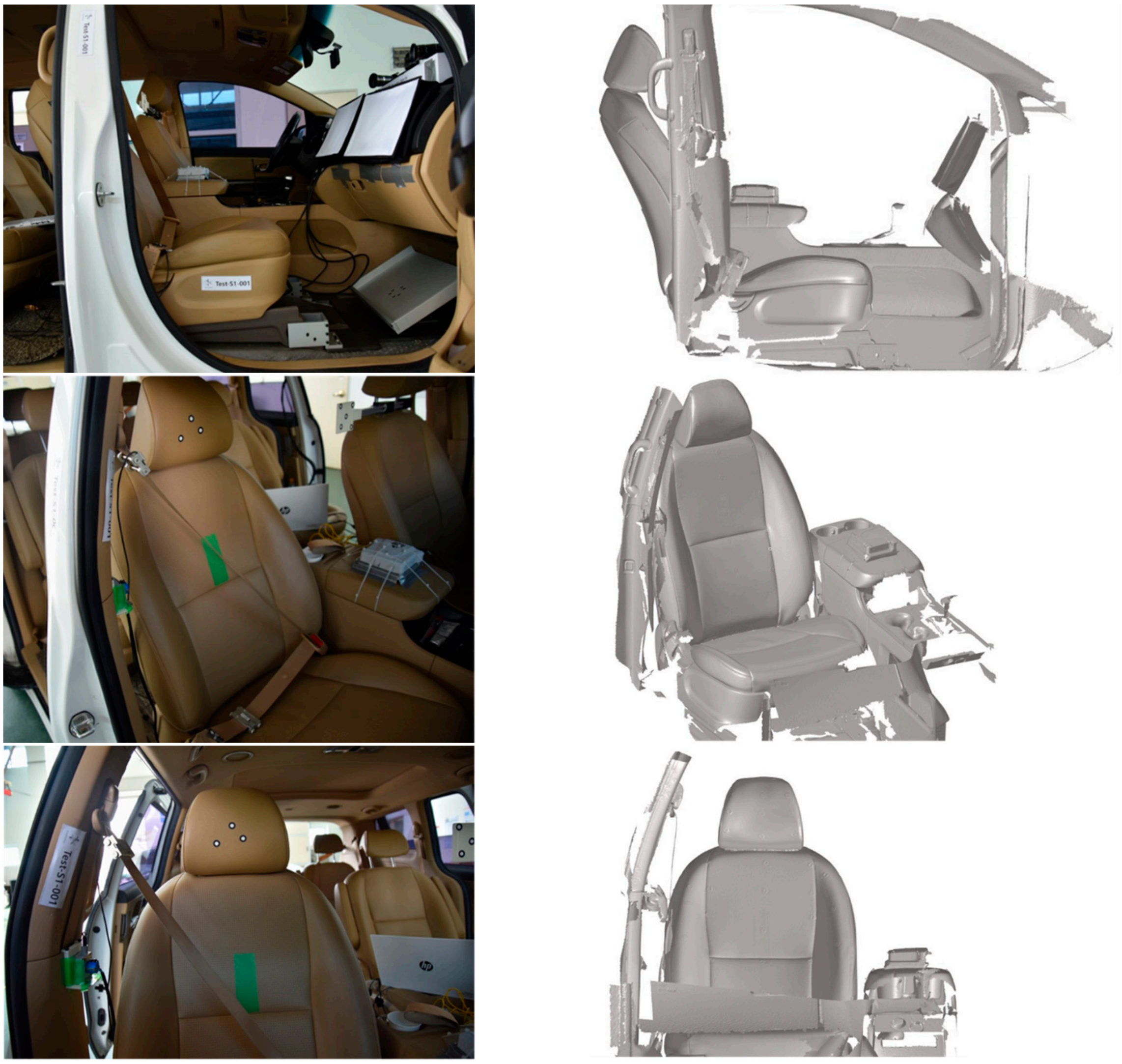

Figure S2.2. Scan data for Upright Seat Test Condition

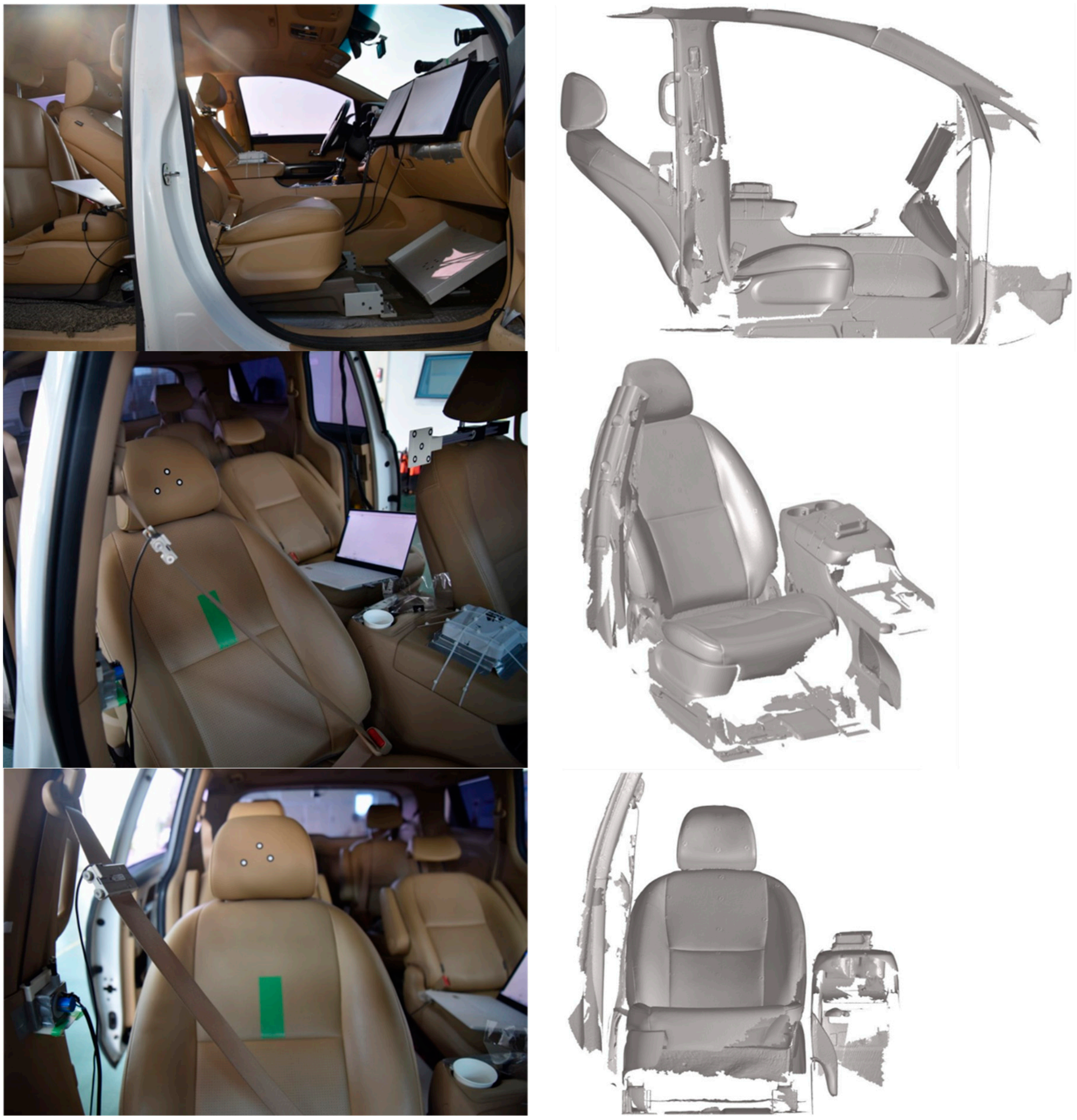

Figure S2.3. Scan data for Recliend Seat Test Condition

1.3. SUPPLEMENTARY S3

1.3.1. Test Setup

To measure the stiffness of the seat, four steel plates, each measuring 250x180x30mm and weighing 10.5kg, were used. The plates were sequentially loaded onto the seat, one to four at a time (Figure S3.1(a)), and each condition was scanned using a 3D scanner to measure the deformation of the seat foam. The stiffness measurements were taken at two positions: the middle of the seat and the location where the occupant would sit (inner position) (Figure S3.1(b)). The scanned results obtained through this process were aligned using Hypermesh, and the deformation of each aligned mesh model was measured, taking into account the 2mm chamfer of the fabricated steel plates. Since the deformation of the seat foam did not appear uniformly, nine reference points were selected on the rigid body to measure the deformation. The deformation was measured by comparing the position of the seat after deformation with the original position of the seat before deformation (Figure S3.2).

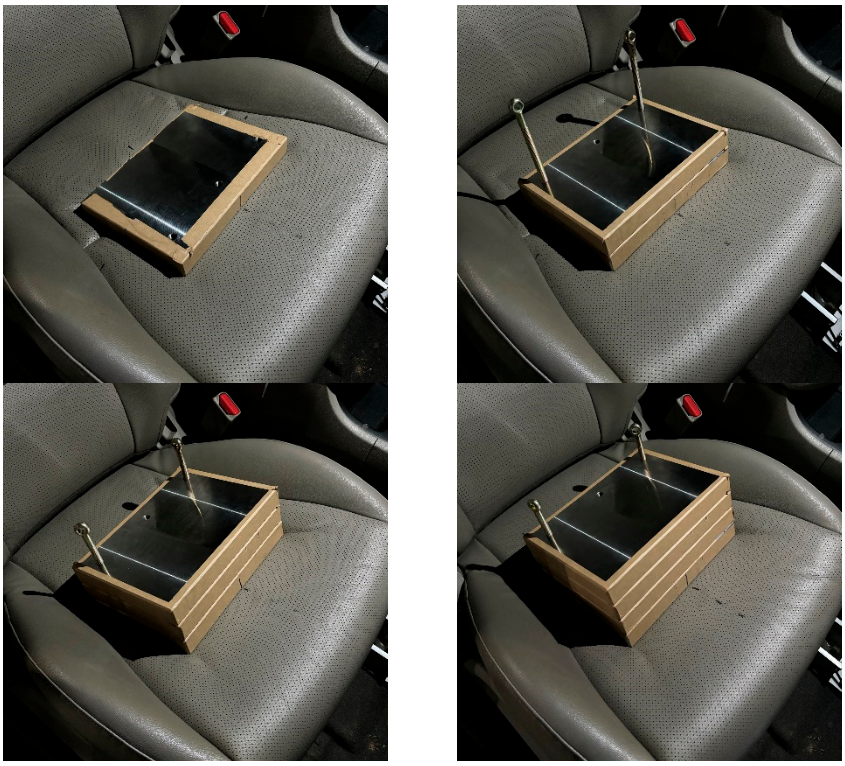

(a)

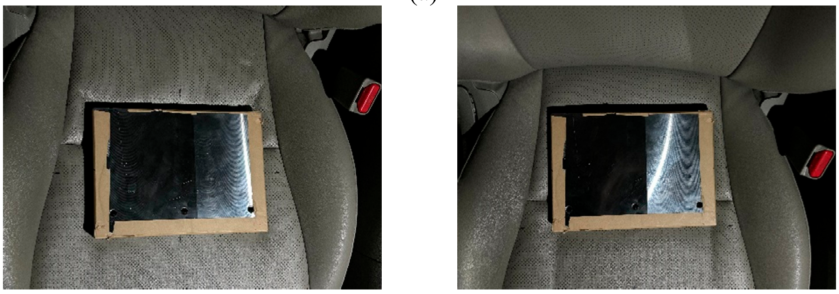

(b)

Figure S3.1. (a) : Seat stiffness test, (b) : Seat test in mid position(left), rearward position(right)

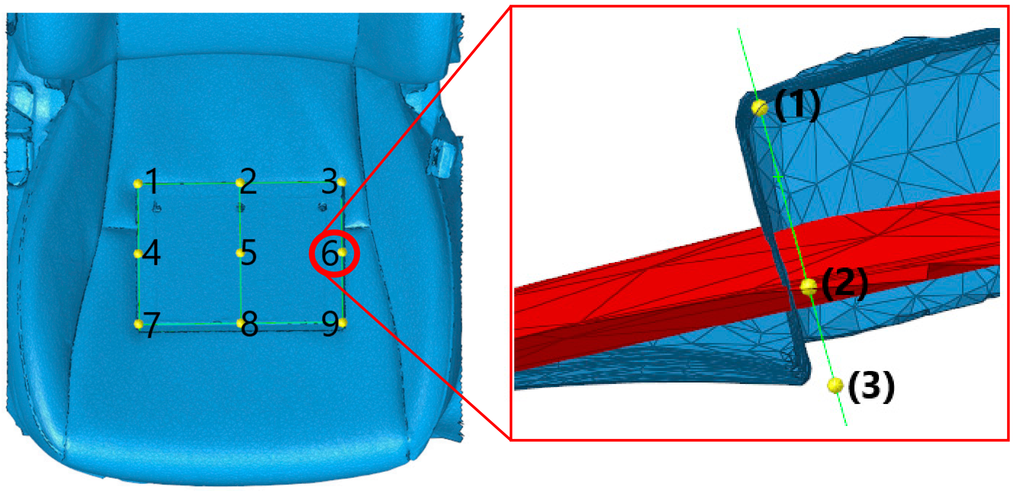

Figure S3.2. Measurement of deformed seat using hypermesh

1.3.2. Result

When checking the average value of the slope of the force-deformation curve, the highest stiffness was measured at 30.02 N/mm at reference point 8 when the rigid body was placed in the middle position, and the lowest stiffness was measured at 8.23 N/mm at reference point 3. Similarly, when the rigid body was placed in the inner position, the highest stiffness was again measured at 17.75 N/mm at reference point 8, while the lowest stiffness was measured at 9.51 N/mm at reference point 3.

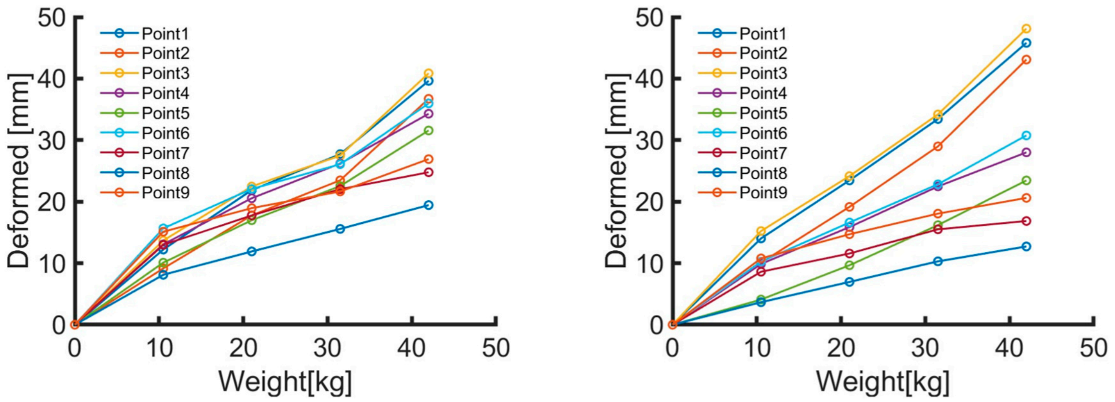

Figure S3.3. Weight-Deformed curve for the seat

1.4. SUPPLEMENTARY S4

The occupant response corridors are presented in the following sections (Figure S4.1 - Figure S4.9). The response corridors were constructed using average and one standard deviation of the responses in each time step.

1.4.1. Braking

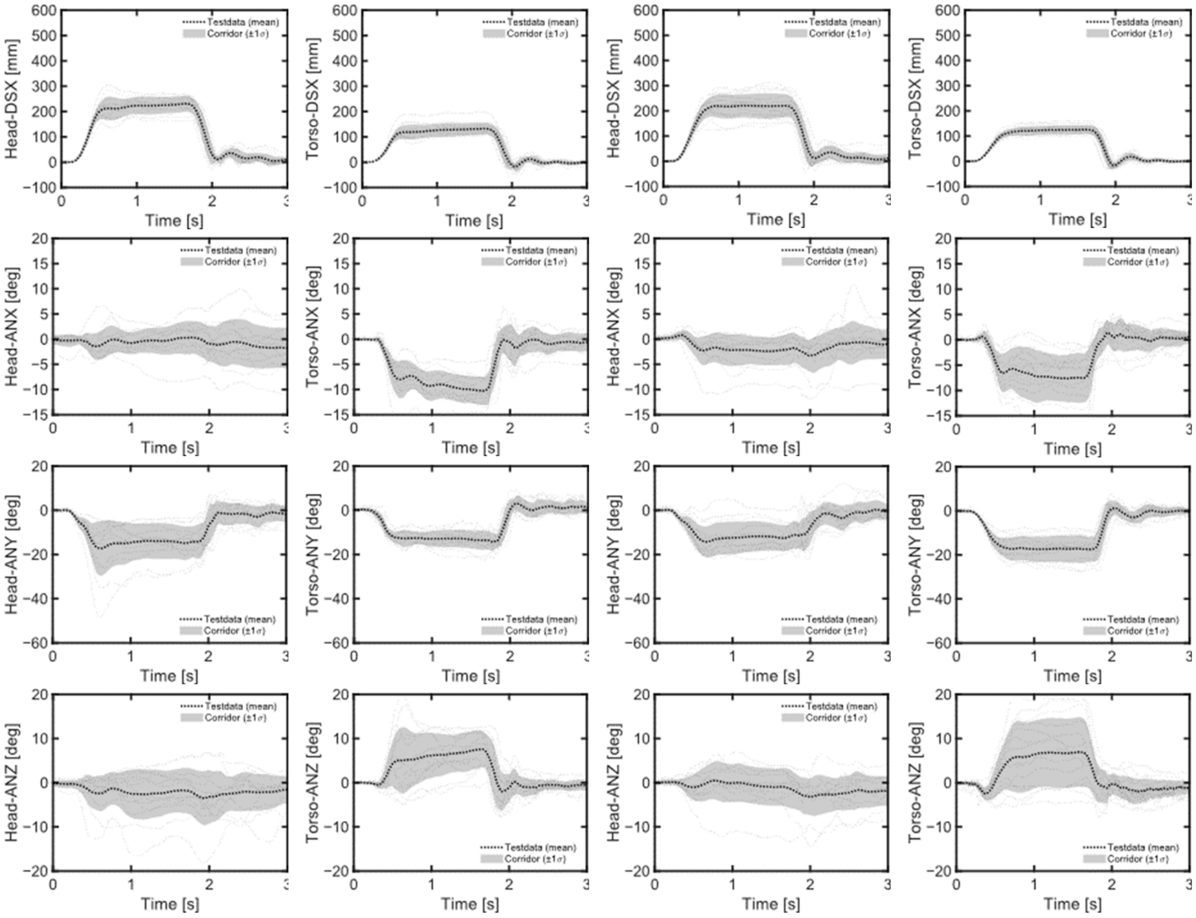

(a) (b)

Figure S4.1. Occupant Response Corridor at Braking Maneuvers : (a) Upright Male (b) Upright Female

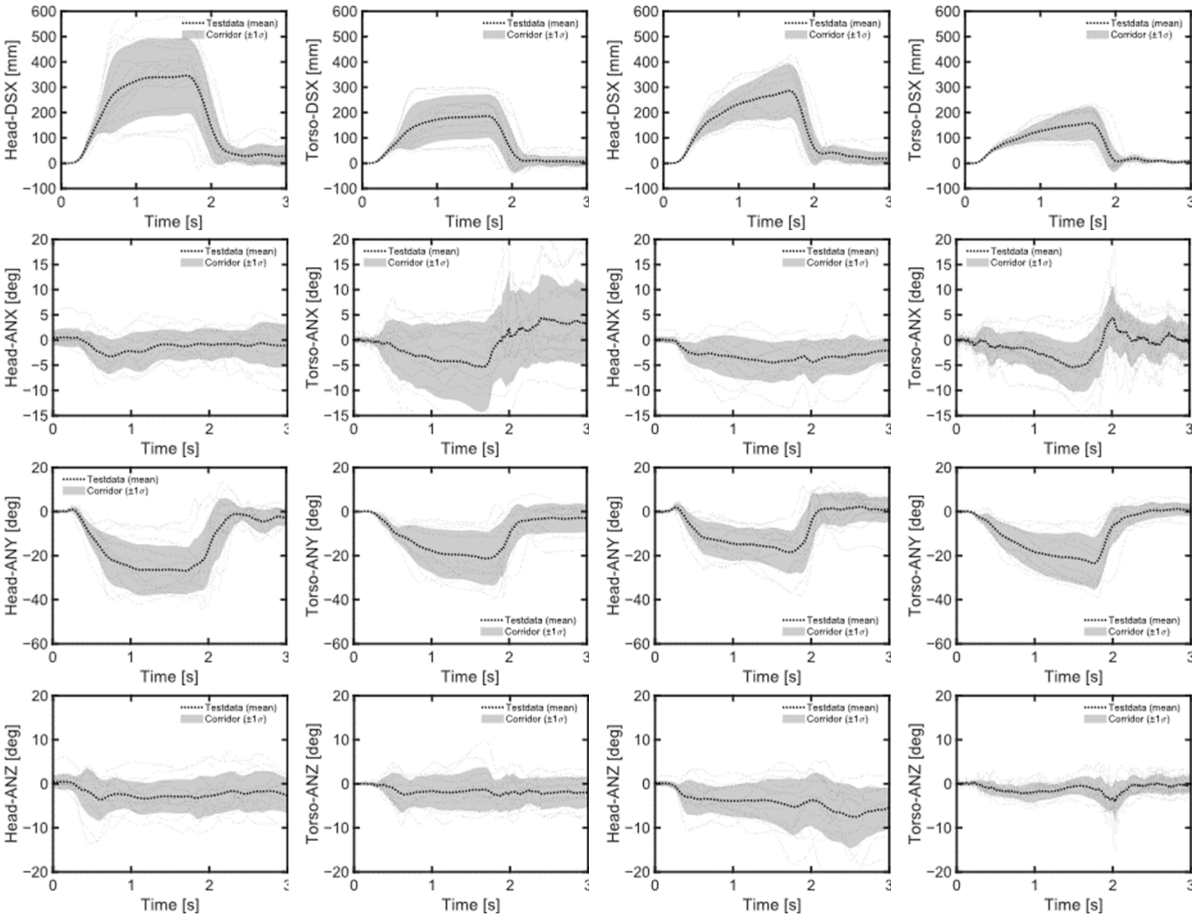

(a) (b)

Figure S4.2. Occupant Response Corridor at Braking Maneuvers : (a) Reclined Male (b) Reclined Female

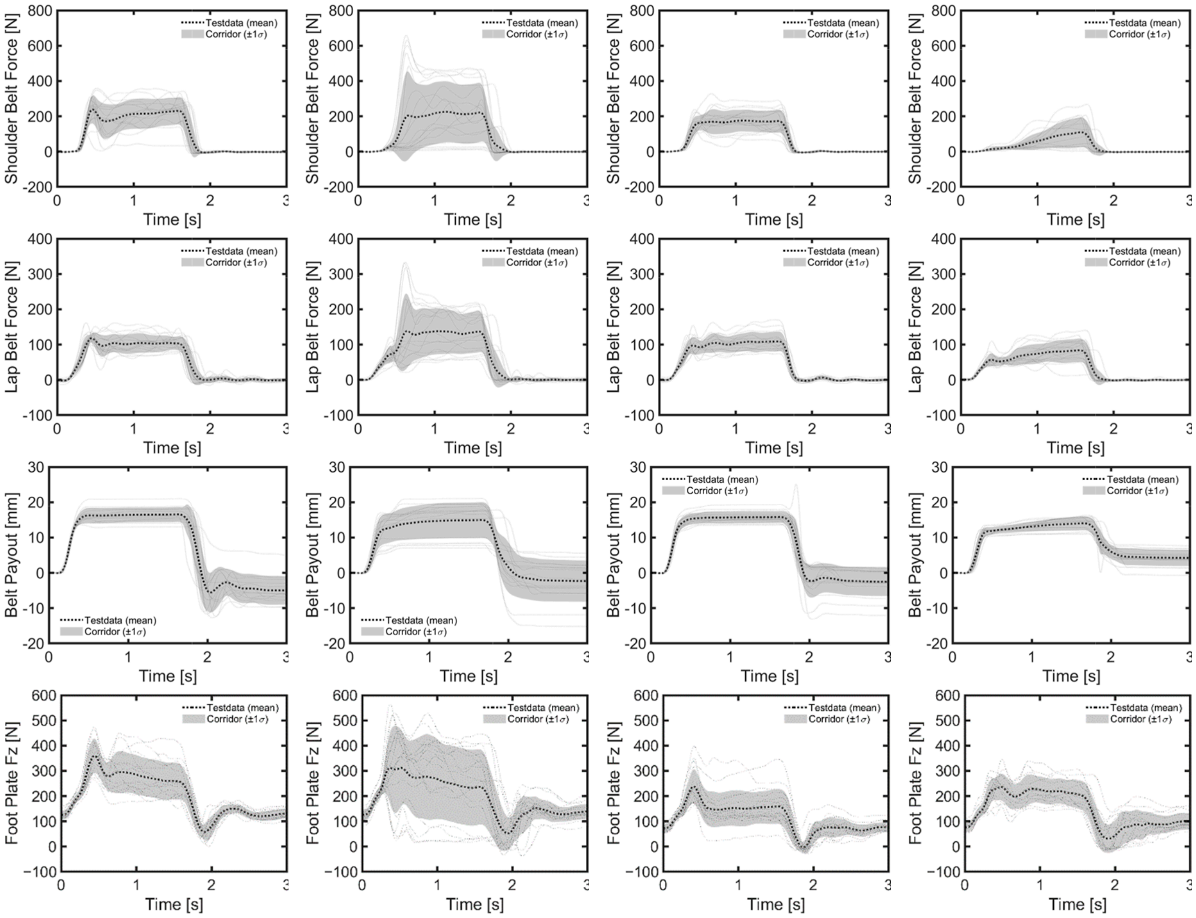

(a) (b) (c) (d)

Figure S4.3. Belt Force and Foot Force measured at Braking maneuvers: (a) Upright Male (b) Reclined Male (c) Upright Female (d) Reclined Female

1.4.2. Left Lane Change

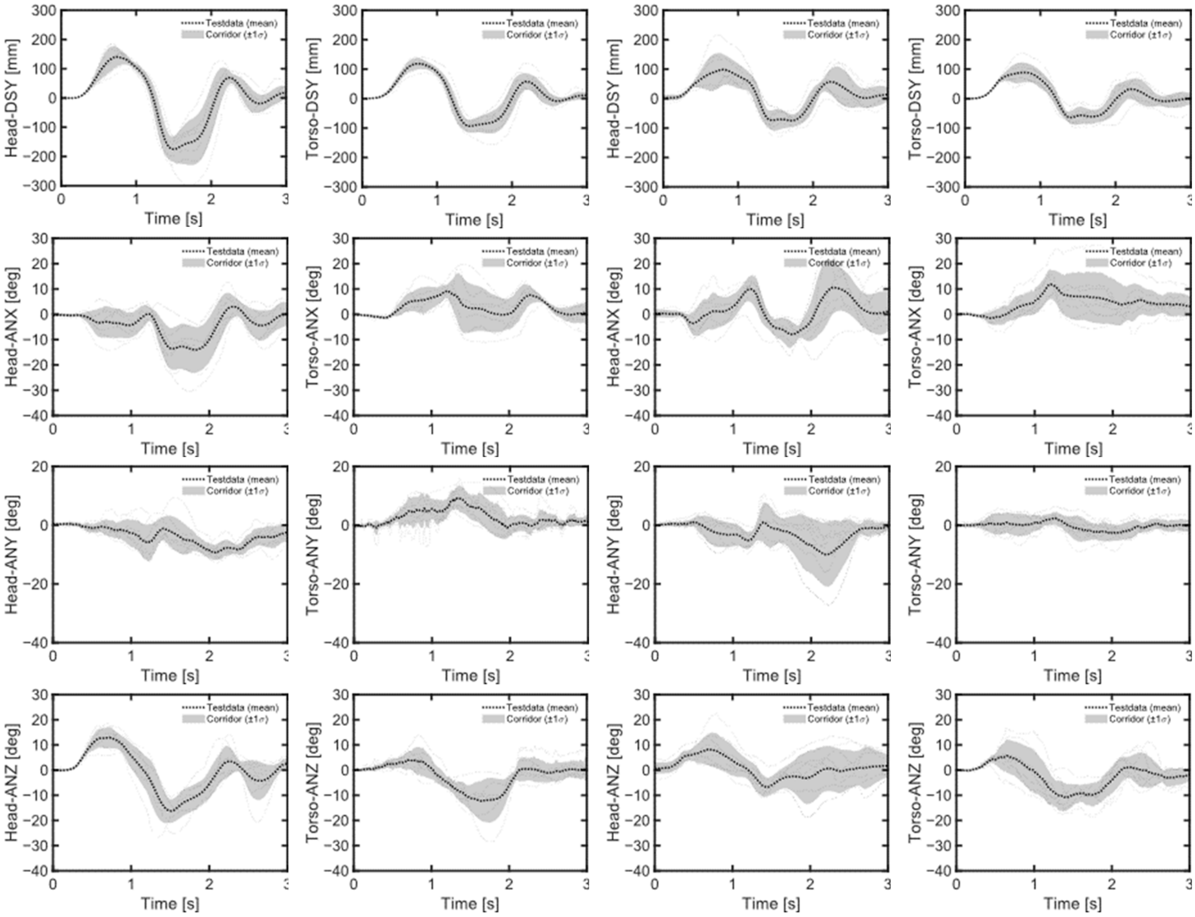

(a) (b)

Figure S4.4. Occupant Response Corridor at Left Lane Change Maneuvers : (a) Upright Male (b) Upright Female

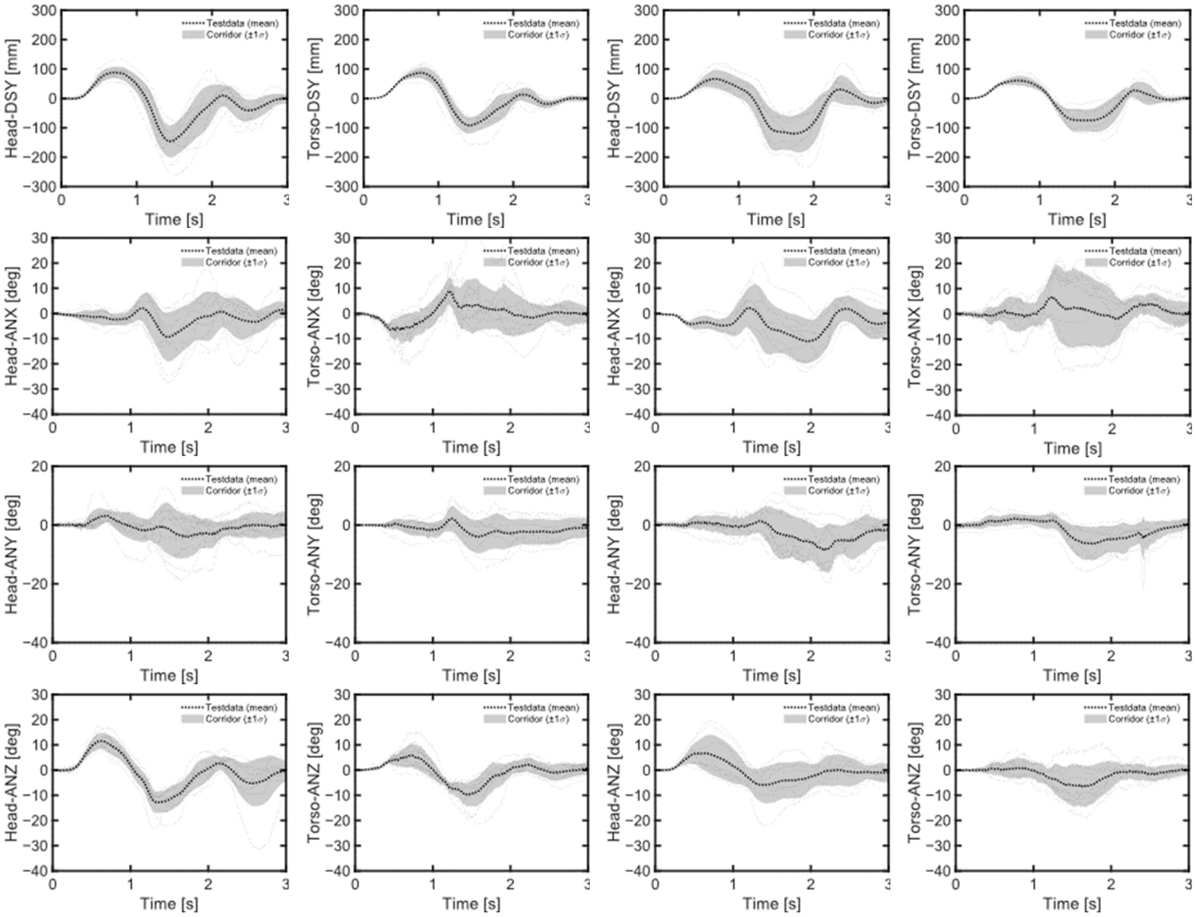

(a) (b)

Figure S4.5. Occupant Response Corridor at Left Lane Change Maneuvers : (a) Reclined Male (b) Reclined Female

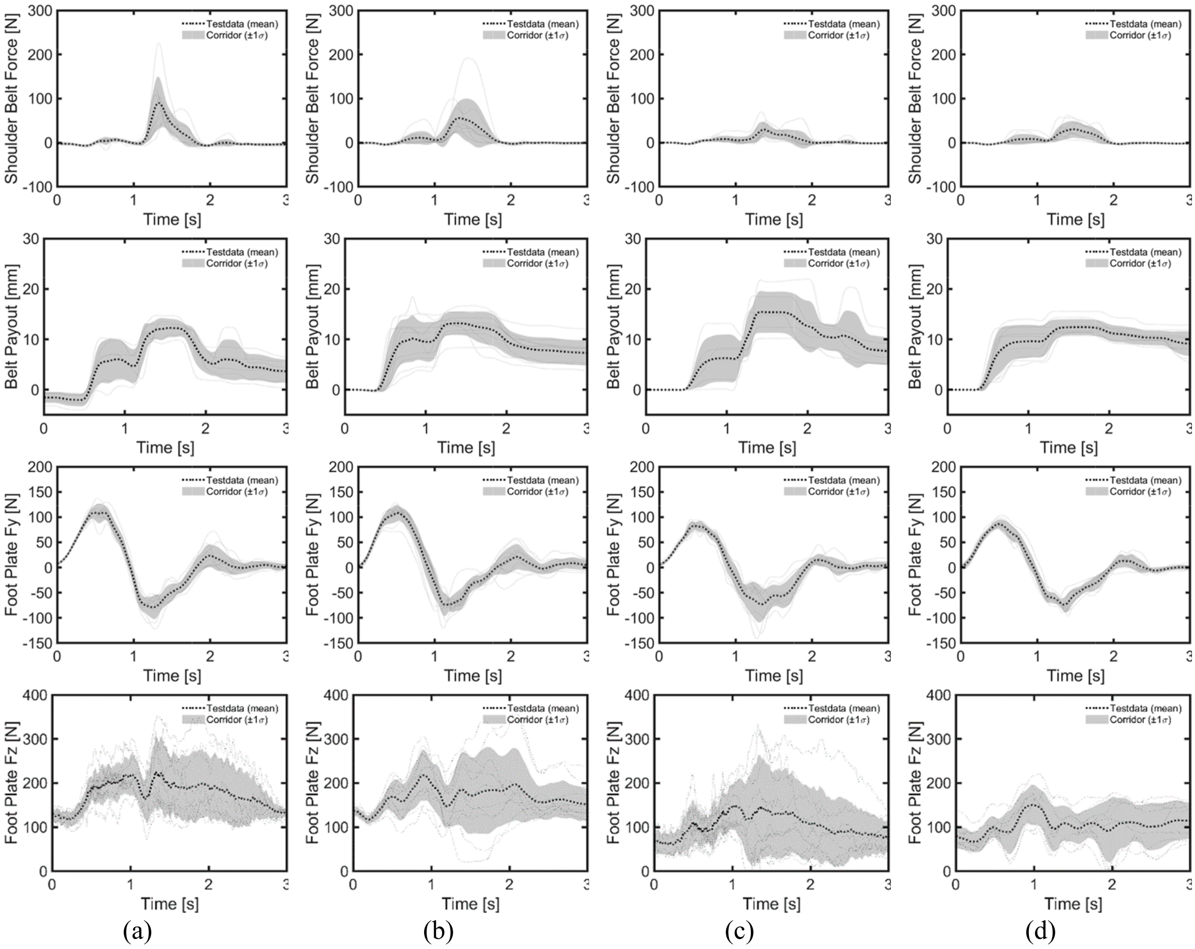

Figure S4.6. Belt Force and Foot Force measured at left lane change maneuvers: (a) Upright Male (b) Reclined Male (c) Upright Female (d) Reclined Female

1.4.3. Right Lane Change

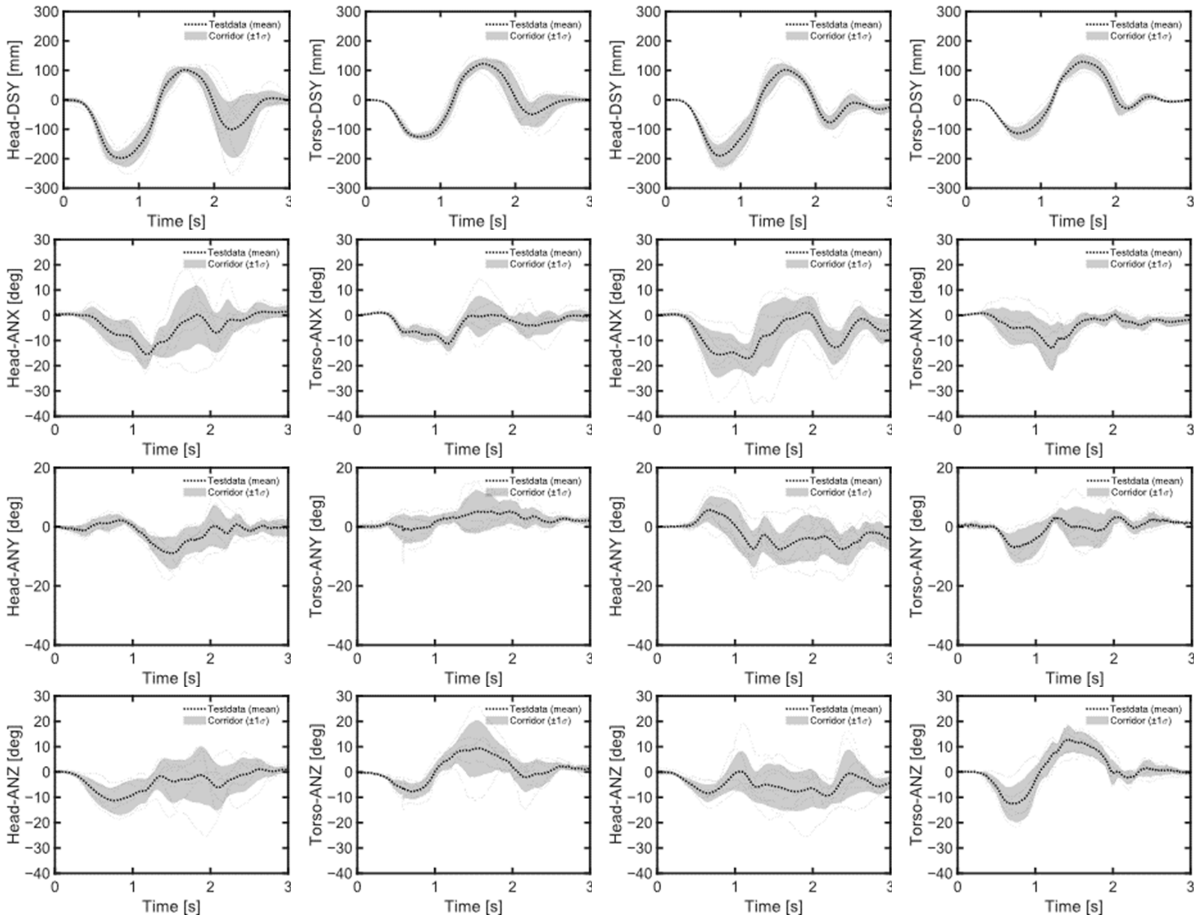

(a) (b)

Figure S4.7. Occupant Response Corridor at Right Lane Change Maneuvers : (a) Upright Male (b) Upright Female

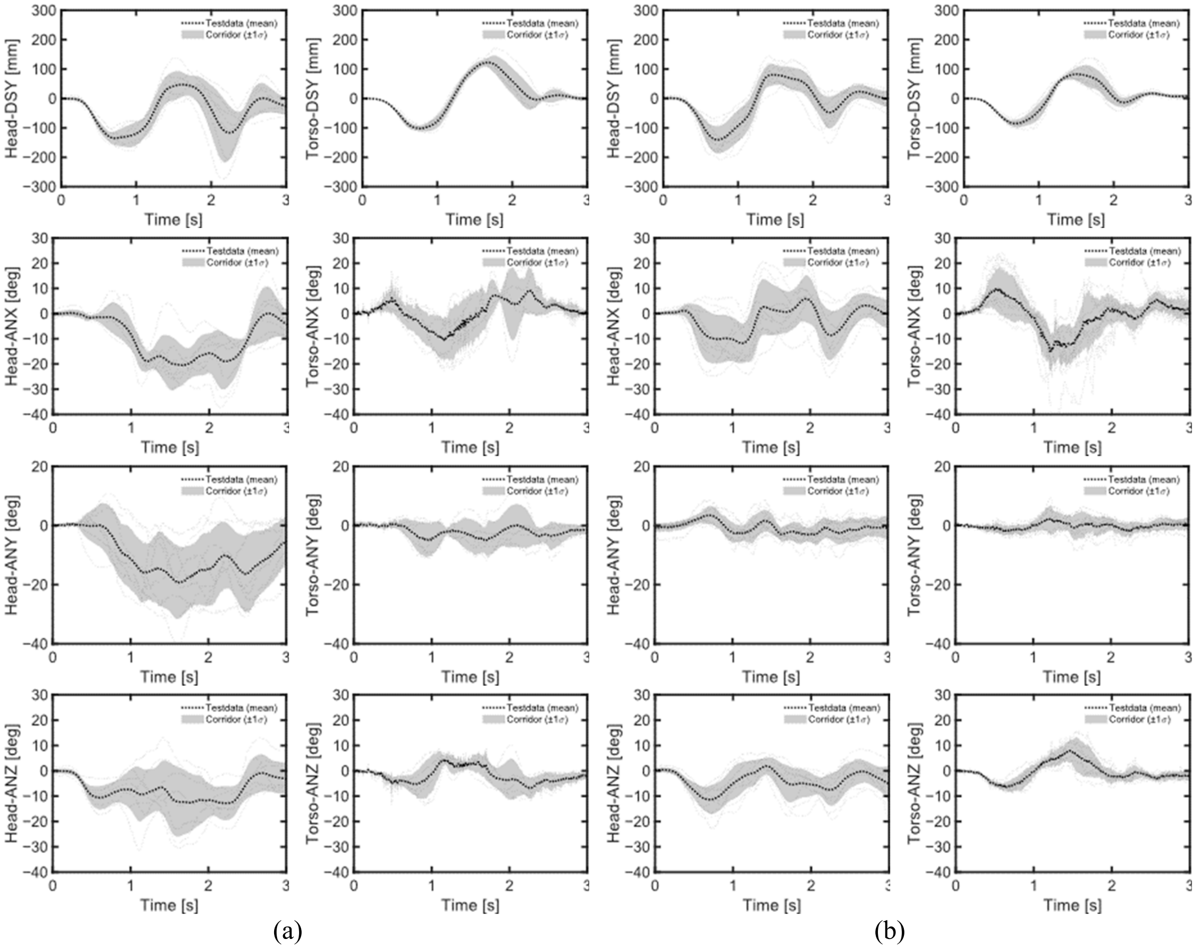

Figure S4.8. Occupant Response Corridor at Right Lane Change Maneuvers : (a) Reclined Male (b) Reclined Female

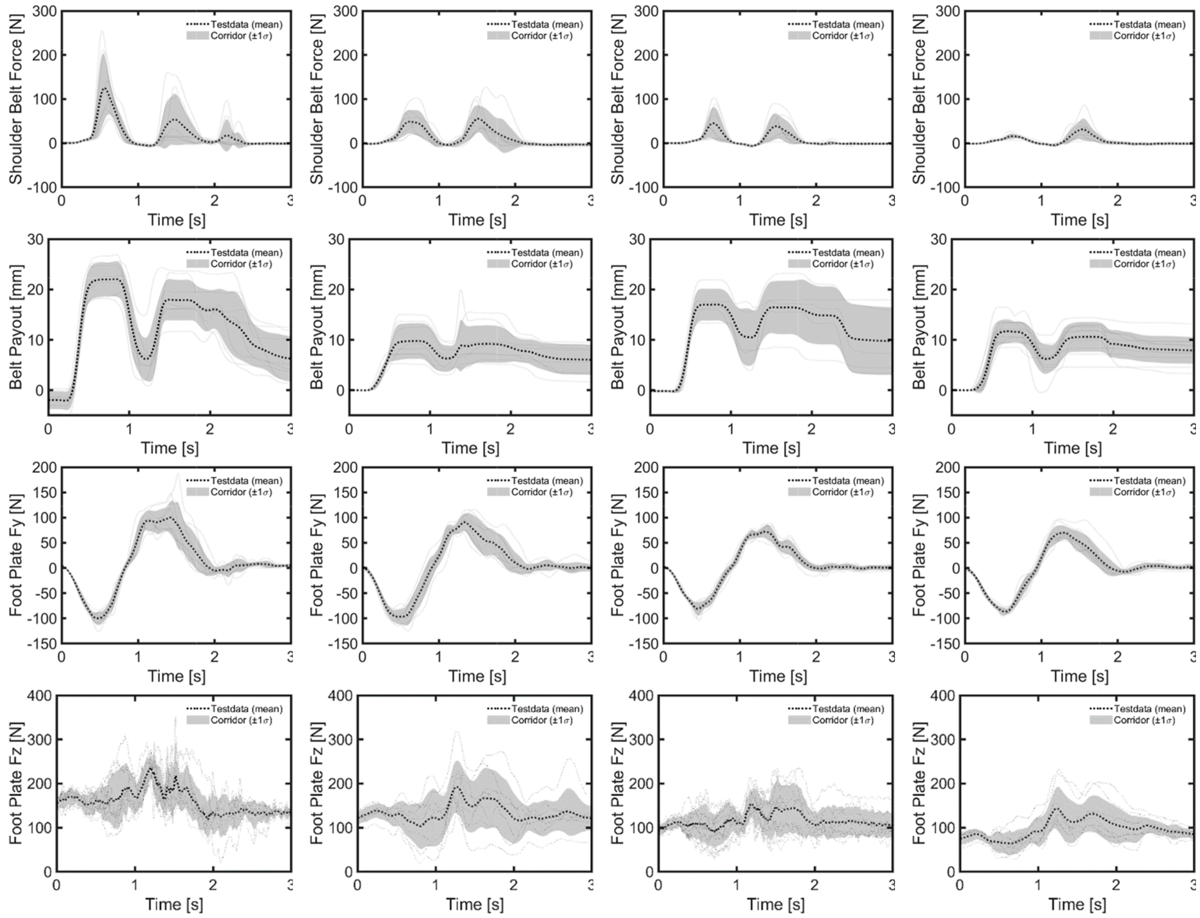

(a) (b) (c) (d)

Figure S4.9. Belt Force and Foot Force measured at right lane change maneuvers : (a) Upright Male (b) Reclined Male (c) Upright Female (d) Reclined Female
